# CNS-border associated macrophages respond to acute ischemic stroke attracting granulocytes and promoting vascular leakage

**DOI:** 10.1186/s40478-018-0581-6

**Published:** 2018-08-09

**Authors:** Jordi Pedragosa, Angélica Salas-Perdomo, Mattia Gallizioli, Roger Cugota, Francesc Miró-Mur, Ferran Briansó, Carles Justicia, Fernando Pérez-Asensio, Leonardo Marquez-Kisinousky, Xabier Urra, Anna Gieryng, Bozena Kaminska, Angel Chamorro, Anna M. Planas

**Affiliations:** 1 Department of Brain Ischemia and Neurodegeneration, Institut d’Investigacions Biomèdiques de Barcelona (IIBB)-Consejo Superior de Investigaciones Científicas (CSIC), Rosselló 161, E-08036 Barcelona, Spain; 2 Àrea de Neurociències, Institut d’Investigacions Biomèdiques August Pi i Sunyer (IDIBAPS), Rosselló 161, E-08036 Barcelona, Spain; 3 Fundació Clínic, Hospital Clínic de Barcelona, Barcelona, Spain; 4 Statistics and Bioinformatics Unit (UEB), Vall d’Hebron Research Institute (VHIR), Barcelona, Spain; 5 Department of Genetics, Microbiology and Statistics, Universitat de Barcelona, Barcelona, Spain; 6 Laboratory of Molecular Neurobiology, Neurobiology Center, Nencki Institute of Experimental Biology, Warsaw, Poland; 7 Present address: Institute of Neuroimmunology and Institute for Multiple Sclerosis Research, University Medical Centre Göttingen, D-37073 Göttingen, Germany

**Keywords:** Perivascular macrophages, Subpial macrophages, Hypoxia, Leukocytes, Brain, Ischemia

## Abstract

The central nervous system (CNS) contains several types of immune cells located in specific anatomic compartments. Macrophages reside at the CNS borders surrounding the brain vessels, in leptomeningeal spaces and the choroid plexus, where they interact with the vasculature and play immunological surveillance and scavenging functions. We investigated the phenotypic changes and role of these macrophages in response to acute ischemic stroke. Given that CD163 expression is a hallmark of perivascular and meningeal macrophages in the rat and human brain, we isolated CD163^+^ brain macrophages by fluorescence activated cell sorting. We obtained CD163^+^ cells from control rats and 16 h following transient middle cerebral artery occlusion, after verifying that infiltration of CD163^+^ peripheral myeloid cells is negligible at this acute time point. Transcriptome analysis of the sorted CD163^+^ cells identified ischemia-induced upregulation of the hypoxia inducible factor-1 pathway and induction of genes encoding for extracellular matrix components and leukocyte chemoattractants, amongst others. Using a cell depletion strategy, we found that CNS border-associated macrophages participate in granulocyte recruitment, promote the expression of vascular endothelial growth factor (VEGF), increase the permeability of pial and cortical blood vessels, and contribute to neurological dysfunction in the acute phase of ischemia/reperfusion. We detected VEGF expression surrounding blood vessels and in some CD163^+^ perivascular macrophages in the brain tissue of ischemic stroke patients deceased one day after stroke onset. These findings show ischemia-induced reprogramming of the gene expression profile of CD163^+^ macrophages that has a rapid impact on leukocyte chemotaxis and blood-brain barrier integrity, and promotes neurological impairment in the acute phase of stroke.

**Electronic supplementary material:**

The online version of this article (10.1186/s40478-018-0581-6) contains supplementary material, which is available to authorized users.

## Introduction

The central nervous system (CNS) contains different subsets of myeloid cells under steady-state conditions. Microglia reside in the brain parenchyma whereas macrophages remain at the CNS borders. CNS border-associated macrophages (BAMs) include cells resident in the perivascular spaces of brain vessels, lining the meninges, and the choroid plexus. BAMs carry out scavenger functions and are fully competent to present antigen to lymphocytes [6, 12, 27, 34, 37]. BAMs derive from hematopoietic progenitors that populate the brain at embryonic stages and are not replaced by bone-marrow derived cells under physiological conditions [18, 19]. Little is known about the function of BAMs under pathological conditions but several lines of evidence support their involvement in the pathogenesis of brain diseases [13]. Perivascular macrophages are protective during bacterial meningitis [44], and participate in the clearance of vascular Amyloid-β (Aβ) deposition in mouse models of Alzheimer’s disease [23, 36, 54]. However, these cells also mediate oxidative stress and cerebrovascular dysfunction induced by Aβ [39]. They contribute to endothelial, neurovascular, and cognitive dysfunction in several models of hypertension [14, 41], and exacerbate the clinical symptoms in a model of experimental allergic encephalomyelitis [43]. A recent study in the mouse using high-dimensional single-cell cytometry and fate mapping identified different subsets of BAMs and showed differential responses of BAMs compared to other brain myeloid cell populations during aging, experimental autoimmune encephalomyelitis, and in a model of Alzheimer’s disease [37].

Ischemic stroke is a frequent disease and one of the main causes of death and permanent disability worldwide [15]. Lack of adequate blood supply to a brain region causes brain damage and triggers sterile inflammation and innate immune responses [5, 26] that are considered as putative targets for therapeutic intervention [4]. While the responses of microglia to ischemic stroke have been extensively studied [33], those of BAMs remain largely unknown. Nevertheless, BAMs might be important in cerebrovascular diseases because they are located at the interface between the blood vessels, the brain parenchyma, and the immune system. In humans [28] and rats [34], perivascular and meningeal macrophages have been classically characterised by the expression of CD163, a membrane scavenger receptor belonging to the cysteine-rich superfamily [31] that is involved in the response to inflammation [10]. In this study, we investigated the phenotype and function of CD163^+^ BAMs in the acute phase of cerebral ischemia/reperfusion in rats.

## Materials and methods

An extended version of the methods is available in the Additional file 1.

### Experimental brain ischemia

Focal brain ischemia was induced in adult male Sprague-Dawley rats (280–320 g body weight) by 1-h intraluminal occlusion of the right middle cerebral artery (MCAo) with reperfusion. Briefly, rats were anaesthetised with isoflurane and cortical cerebral perfusion was measured with a laser–Doppler flowmeter (PF4001 Master, Perimed). Body temperature was maintained at 37.5 °C during surgery with a heating blanket connected to a rectal probe. The MCA was occluded with a filament (Doccol #403912PK10Re). Rats were excluded from the study if the mean drop in cerebral perfusion during ischemia did not reach at least 65% of the basal value. Overall mortality after ischemia was <10%. A neurological test on a nine-point scale (0 = no deficit to 9 = highest handicap) was performed at 24 h. We scored: (i) spontaneous activity (moving/exploring = 0, moving without exploration = 1, no moving or only when pulled by the tail = 2); (ii) circling to the left (none = 0, circling when elevated by the tail and pushed or pulled = 1, spontaneous circling = 2, circling without displacement = 3); (iii) resistance to left (contralateral) forepaw stretching (full resistance = 0, rats offer some resistance but they allow stretching = 1, rats offer no resistance =2), and (iv) the parachute reflex (symmetrical = 0, asymmetrical = 1, contralateral forelimb retracted = 2). The brain was imaged 24 h after MCAo with MRI in a 7.0 T horizontal animal scanner (BioSpec, Bruker BioSpin, Ettlingen, Germany). Brain lesions were evaluated by T2 mapping and lesion volume was measured with ImageJ.

### Drug administration

Anesthetized rats (isoflurane) received an i.c.v. injection of 30 μl liposomes containing either clodronate (5 μg/μl), or PBS as the vehicle (ClodronateLiposomes.com, Haarlem, The Netherlands) in the left ventricle. Treatment was randomly allocated according to a randomization list generated at the beginning of each set of experiments. Each treatment received a code that did not reveal its identity. Drug administration and all further interventions and measurements were carried out in a blinded fashion. Ischemia was induced 4 days after drug administration.

### Flow cytometry

Fresh brain tissue was processed with the Neural Tissue Dissociation Kit (P) (#130–092-628, Miltenyi Biotec). A 30–0% percoll gradient was used to remove myelin and cell debris to obtain a single-cell suspension. The pellet was washed and stained with the life/death fixable cell staining Aqua (ThermoFisher Scientific), and cells were immunostained with anti-CD11b (clone OX-42, Alexa Fluor647; AbDSerotec or PerCP-Cy5.5; BioLegend) at 1:40 dilution, anti-CD163 (clone ED2, FITC or PE, AbdSerotec) diluted 1:20, anti-CD45 (clone OX-1 labelled with PE-Cy7, BioLegend, or Alexa Fluor 488 AbdSerotec) diluted 1:50, anti-granulocytes (clone REA535, APC-Vio770, Miltenyi Biotec) diluted 1:50, anti-CD3 (clone G4.18, PE, BD Pharmingen) diluted 1:100, anti-CD4 (clone OX-35, BV711, BD Biosciences) diluted 1:200, anti-CD8 (clone OX-8, Vioblue, Miltenyi-Biotec) diluted 1:200, anti-CD161 (clone 3.2.3, APC, Biolegend) diluted 1:200, anti-γδTCR (clone V65, APC-Vio770, Miltenyi-Biotec) diluted 1:100, and anti-CD25 (clone OX-39, FITC, BD Pharmingen) diluted 1:100. We used Flow-count Fluorospheres (Beckman-Coulter) for absolute cell counting. Data were acquired in a BD LSRFortessa SORP flow cytometer (BD Biosciencies) using the BD Diva software (BD Biosciences) and were analysed with FlowJo v10 software (FlowJo).

### Cell sorting

CD163^+^ macrophages and microglia were isolated from the control rat brain and from the brain 16 h post-ischemia using fluorescence activated cell sorting (FACS). Briefly, the right brain hemisphere was processed with the Neural Tissue Dissociation Kit and a percoll gradient, as described above for flow cytometry, and single cells were immunostained with CD11b and CD163. CD11b^+^CD163^+^ cells corresponding to brain resident macrophages and CD11b^+^CD163^−^ cells were collected in RNAse-free PBS using Aria II cell sorter (BD Biosciences). We verified the purity of the sorted cell populations by flow cytometry in independent experiments.

### RNA extraction

RNA was extracted from samples of FACS-sorted CD163^+^ macrophages and FACS-sorted CD163^−^ microglia with PureLink™ RNA Micro Kit (#12183016, Invitrogen). On-column DNAse step was performed to avoid genomic DNA contamination. RNA purity was assessed by RNA Pico Chip BioAnalyzer 2100 (Agilent). RNA was also extracted from brain tissue samples with the PureLink™ RNA Mini Kit (#12183018A, Invitrogen) using Trizol® Reagent (Life Technologies). In this case, we assessed the RNA quantity and quality using a ND-1000 micro-spectrophotometer (NanoDrop Technologies).

### qRT-PCR

Total RNA was reverse-transcribed using a mixture of random primers (High Capacity cDNA Reverse Transcription kit, Applied Biosystems). For brain tissue, 1000 ng of total RNA were reverse-transcribed and the final product was diluted 6 times in RNAse-free water. For RNA of sorted cells, cDNA was pre-amplified (TaqMan® Pre Amp Master Mix (2×) #4384266) with a pool of TaqMan probes, and the final pre-amplified product was diluted 20 times with tris-EDTA (TE) buffer pH 8.0 (#BP2473, Fisher Bioreagents). Quantitative real-time RT-PCR analysis was carried out with Taqman system (#4304437, Life Technology) using the iCycler iQTM Multicolor Real-Time Detection System (Bio-Rad, Hercules, CA, USA).

### RNA microarray and bioinformatics

Six RNA samples obtained from sorted CD11b^+^CD163^+^ cells (from three control rats and three ischemic rats at 16 h post-ischemia) with RIN values> 9.2 were selected for RNA microarray study with GeneChip Rat Genome 230 2.0 Array, 3’IVT Pico reagent kit (Affymetrix). The images were processed with the Expression Console software (Affymetrix) to check the array quality. Genes selected as being differentially expressed were clustered to look for common patterns of expression. Bioinformatics analyses of functional and biological significance were carried out.

### Immunohistochemistry of paraffin embedded brain sections

Rats were anesthetized with isoflurane and perfused through the heart with saline followed by 4% PFA. Careful extraction of the brain from the skull allowed keeping most of the pia meningeal layer attached to the brain tissue. The brain was kept in 4% PFA overnight at 4 °C, washed in phosphate buffer and embedded in paraffin. Immunohistochemistry was carried out in 5 μm-thick paraffin section with the following primary antibodies: a mouse monoclonal antibody against CD163 (clone ED2, which recognizes the rat CD163 cell surface glycoprotein [7]) (# MCA342GA, 0.5 mg/mL, AbD Serotec, Bio-Rad) diluted 1:50, and rabbit polyclonal antibodies against Iba-1 (# 019–19,741, 0.5 mg/mL, Wako Chemicals USA, Inc.) diluted 1:500, and against myeloperoxidase (MPO) (# A0398, 3.3 mg/mL, Dako), diluted 1:100, using the EnVisionTM Detection System; (# K5007, Dako). Following incubation with biotinylated secondary antibodies (anti-rabbit made in goat # BA-1000; and anti-mouse made in horse # BA2001; both from VECTOR and used at 1:200 dilution), the immunoreaction was visualized with the avidin-biotin peroxidase method (ABC, Vector, Palex Medical S.A., Sant Cugat del Vallès, Spain) and diamenobenzidine. Cell counting was performed in a blinded fashion.

### Evans blue technique

A solution of 2% Evans blue (Sigma-Aldrich) (*w*/*v* in saline) was administered i.v. (4 mL/kg of body weight) 22 h after ischemia. Two hours later, rats were anesthetized and perfused with saline. We obtained images of 1-mm thick coronal sections of fresh brain that were analysed with ImageJ software.

### Western blotting

Proteins were separated by electrophoresis in 12% polyacrilamide gels and were transferred to polyvinylidene fluoride membranes (Immobilon-P, # IPVH00010, MilliporeSigma) and incubated overnight at 4 °C with a goat polyclonal antibody anti-VEGF-A (#AF564, R&D) diluted 1:500, followed by a secondary anti-goat HRP antibody diluted 1:2000. β-tubulin was used as the loading control.

### Post-mortem tissue of stroke patients

We studied the brain of three stroke patients (female; 63, 81 and 89 years old) deceased on day 1 after ischemic stroke onset. Two patients had right MCA infarcts and the third had a vertebro-basilar infarction, with a National Institute of Health Stroke Scale (NIHSS) severity score of 20, 13, and 9, respectively. Only the first of these patients received mechanical thrombectomy, whereas the other two patients did not receive any revascularization therapy. None of the patients received tPA. The mean ± SD time lapse from exitus to necropsy was 4.3 ± 3.2 h. Expert neuropathologists obtained ischemic tissue that was embedded in optimal cutting temperature (OCT) compound and immediately frozen in liquid nitrogen for later sectioning in a cryostat at 5 μm. The sections were processed for immunofluorescence using the following primary antibodies: mouse monoclonal antibody anti-CD163 (clone EDHu-1, 1 mg/mL, # MCA1853, Serotec, Bio-Rad) diluted 1:200; rabbit polyclonal antibody anti-VEGFA (0.9 mg/mL, # ab46154, Abcam) diluted 1:100; and sheep polyclonal antibody anti-vWF (# ab11713, 6.8 mg/mL, Abcam) diluted 1:100. Sections were incubated overnight at 4 °C with primary antibodies followed by incubation for 2 h at room temperature with secondary antibodies (Life Technologies): Alexa Fluor-488 (# A11017 anti-mouse made in goat, and # A21206 anti-rabbit made in donkey), Alexa Fluor-546 (# A10036 anti-mouse made in donkey, and # A10040 anti-rabbit made in donkey) and Alexa Flour-647 (# A21448 anti-sheep made in donkey), all diluted 1:500. 0.3% Sudan black in 70% ethanol was used to reduce tissue autofluorescence. Immunoreaction controls were carried out by omission of the primary antibodies, and by substituting the primary anti-VEGFA antibody by rabbit immunoglobulin fraction (# 0903, 20 mg/mL, Dako) diluted 1:2200. Sections were counterstained with 4′,6-diamidino-2-phenylindole (DAPI) (Invitrogen) to visualize the cell nuclei and they were observed under a confocal laser microscope (Zeiss LSM 880). Confocal images were processed with ImageJ software to display the pictures.

### Statistical analyses

Comparisons between two groups were carried out with two-tailed Mann-Whitney or unpaired Student t-test after testing for normal distribution using the Kolmogorov-Smirnov test. Multiple comparisons were carried out with the non-parametric Kruskal-Wallis test followed by post-hoc Dunn’s test. Two-way ANOVA was used to compare group differences by treatment (clodronate vs. vehicle) and condition (ischemia vs. control). The Bonferroni’s Multiple Comparison tests was used for post-hoc analysis. Statistical analyses were carried out with GraphPad Prism software. The sample size in experiments designed to investigate the effect of drug treatment on stroke outcome was *n* = 16, as calculated using G*power 3.1 software (Düsseldorf University) with an estimated effect size d of 0.9, alpha level of 0.05, and statistical power of 0.8.

## Results

### Features of CD163^+^ cells in the control brain and in the acute phase of stroke

**Fig. 1:**
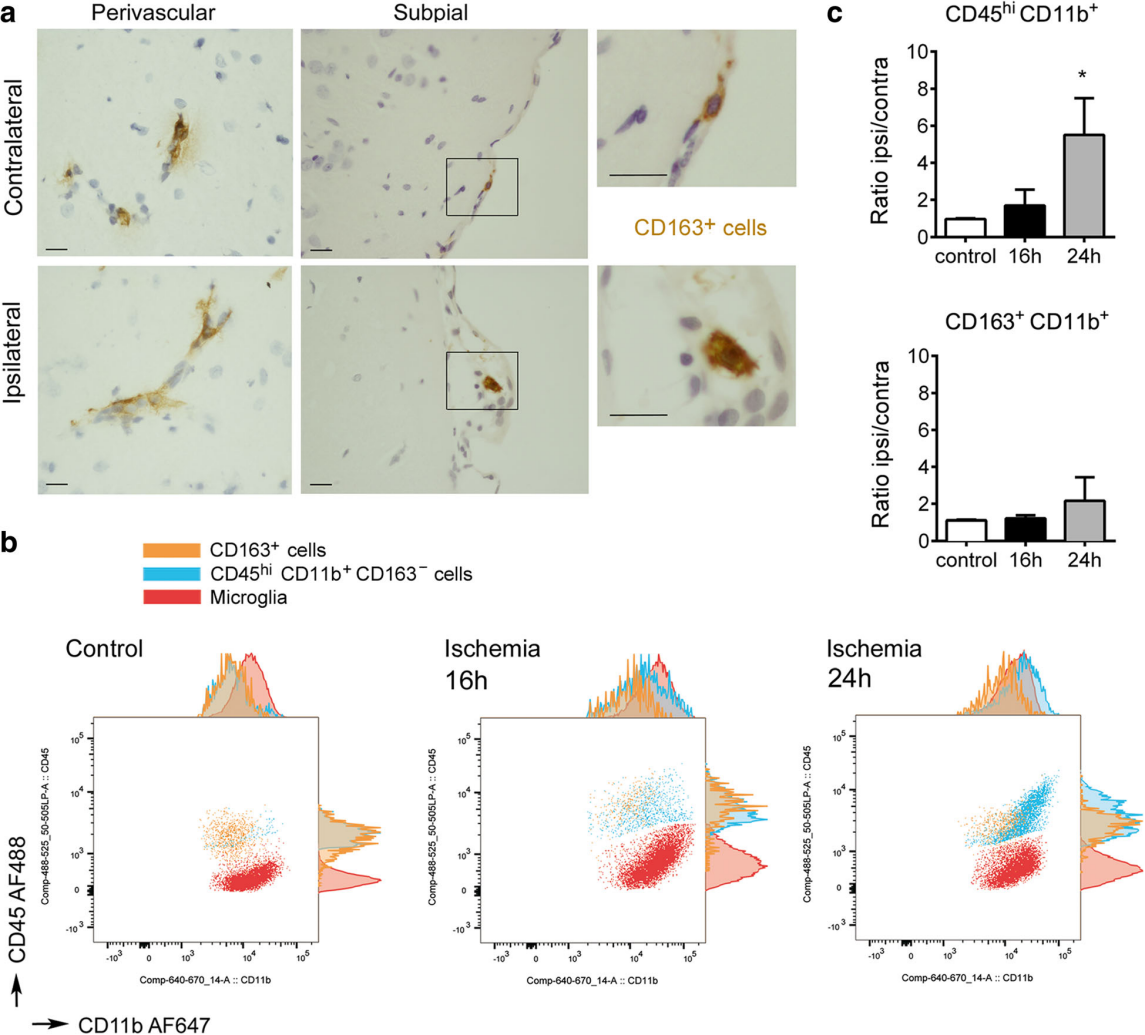
Features of the CD163^+^ macrophages in the acute phase of stroke. **a**) CD163 immunohistochemistry in paraffin brain sections 8 h after MCAo shows CD163^+^ cells (brown) in perivascular and subpial spaces of the contralateral and ipsilateral hemispheres. Panels in the right are magnifications of the squares in the adjacent panels. Images are representative of four rats. Scale bar = 10 μm. **b**) Flow cytometry of myeloid cells in the control (*n* = 2) and ischemic brain tissue 16 h (*n* = 4) and 24 h (*n* = 7) after MCAo. The population of CD163^+^ cells (orange) is maintained after ischemia, but the population of CD45^hi^CD11b^+^ cells (blue) progressively increases due to infiltration of peripheral myeloid cells to the ischemic tissue. Microglial cells (CD45^low^CD11b^+^) are shown in red. **c**) Quantification of the brain myeloid cell populations within all live cells by flow cytometry. For each animal, we calculated the fold increase in the ischemic (ipsilateral, ipsi) hemisphere versus the contralateral (contra) hemisphere. As expected, the ratio between the right/left hemispheres in control rats was equal to 1 (mean±SD, 0.965 ± 0.05 for CD45^hi^CD11b^+^ cells, and 1.115 ± 0.05 for CD163^+^ cells). The ratio ipsi/contra progressively increased after ischemia for CD45^hi^CD11b^+^ CD163^−^ cells (*p < 0.05, Kuskall-Wallis test followed by post-hoc Dunn’s test). In contrast, the ratio ipsi/contra for CD163^+^ cells was similar to controls at 16 h and the increases at 24 h were very small and not statistically significant. Values in the graph are expressed as the mean and SD of the indicated number of rats per group

The CD163^+^ rat brain cells studied in this work include perivascular macrophages and subpial macrophages that remained attached to the glial limitans after removing the brain from the skull (Fig. 1a). Flow cytometry of control brain tissue showed a higher level of CD45 expression in CD163^+^ macrophages than in microglia (CD45^low^CD11b^+^ cells) (Fig. 1b). Ischemia induces macrophage infiltration to the brain peaking after several days [17, 47, 49, 52, 53]. We examined the CD163^+^ cells and the CD45^+^CD11b^+^ myeloid cells in the control brain tissue and the brain tissue at 16 h and 24 h post-ischemia. Following ischemia, the population of CD45^hi^CD11b^+^ myeloid cells progressively increased. In contrast, the population of CD163^+^ cells remained similar to controls at 16 h and showed a tendency to increase in the ipsilateral hemisphere at 24 h (Fig. 1b,c). These results showed that most peripheral myeloid cells infiltrating the brain tissue in the first hours post-ischemia did not express CD163.

### Ischemia-induced changes in the gene expression profile of CD163^+^ BAMs

**Fig. 2:**
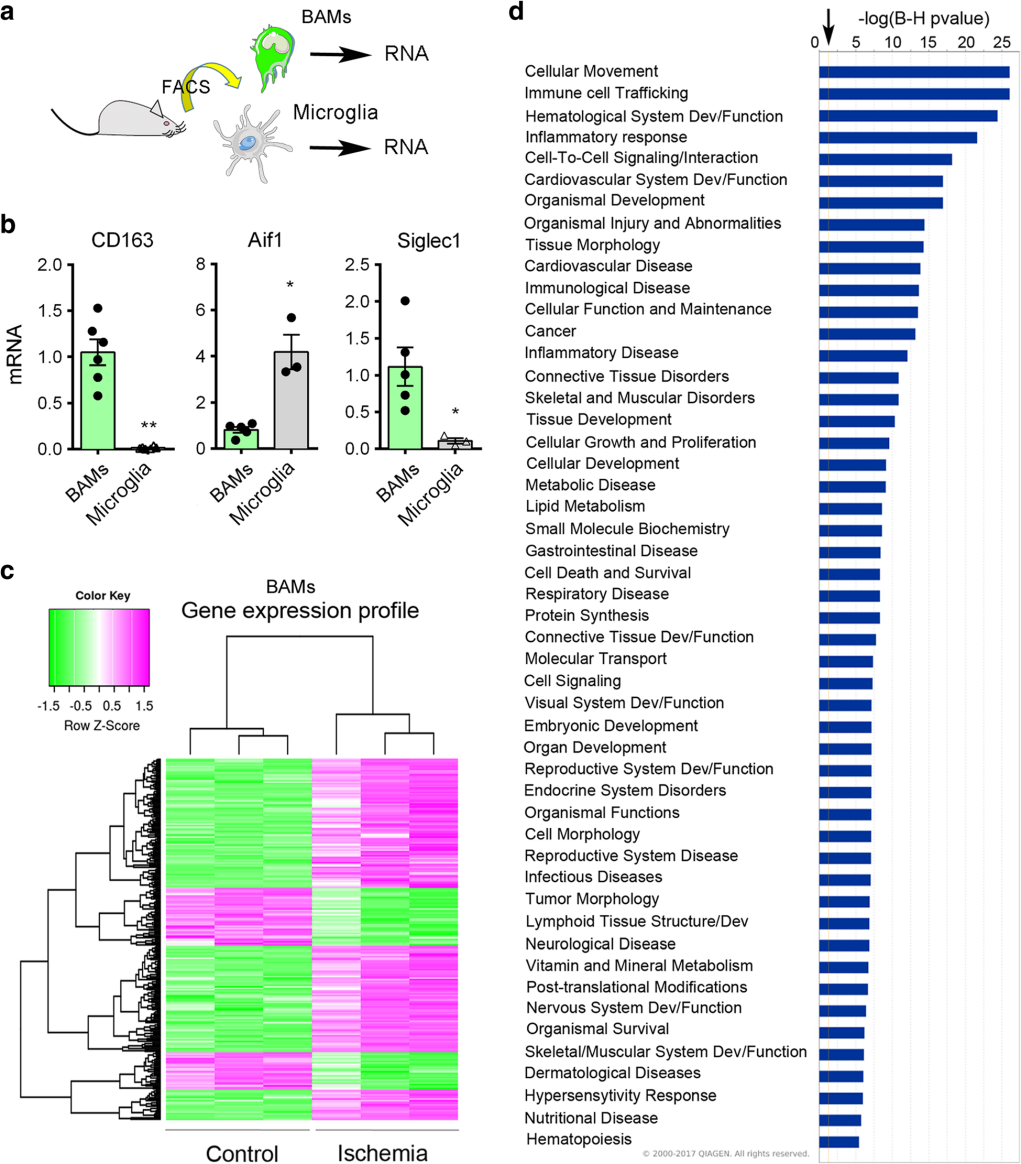
Gene expression profile of CD163^+^ macrophages after brain ischemia. **a**) We isolated CD11b^+^CD163^+^ BAMs and CD11b^+^ CD163^−^ microglia by FACS from control rat brain and obtained RNA for gene expression analysis. Colors for cells in the drawings are arbitrary. **b**) By qRT-PCR we validated that sorted macrophages, but not sorted microglial cells, express *Cd163*. The expression of *Aif1* (Iba-1) is higher in microglia than macrophages, whereas the expression of *Siglec1* (CD169) is lower in microglia. Values are expressed as fold versus the mean value of CD163^+^ macrophages and are the mean±SD of *n* = 3–6 samples per group. **p < 0.01, *p < 0.05, two-tailed Mann-Whitney test. **c**) RNA was extracted from CD163^+^ cells immunosorted from the control and the ischemic rat brain at 16 h of reperfusion (*n* = 3 per group) to study ischemia-induced changes in gene expression profile using Affymetrix microarrays. The global heat map, where each lane represents macropahge gene expression from the brain of different control or ischemic rats, shows results of the microarray analysis with∣logFC∣> 2 and FDR < 0.01. **d**) Top diseases/functions associated to gene expression profile changes were obtained with ingenuity pathway analysis (IPA) gene ontology algorithms showing category scores. The minimum significance level (−log(*p*-value)) from Fisher’s exact test is indicated by the threshold (arrow). Inflammatory and immune functions are highlighted suggesting the involvement of CD163^+^ macrophages in such responses after brain ischemia

To characterize the general phenotype of CD163^+^ macrophages and compare it with that of microglia, we isolated the CD163^+^ cells and the CD163^−^ microglia from the control rat brain by fluorescence-activated cell sorting (FACS) (Fig. 2a, see gates used for cell sorting in Additional file 1: Figure S1a). We checked the purity (mean ± SD, n rats) of the sorted cells by flow cytometry and found that 92.0 ± 4.2% (*n* = 4) of the sorted cells were CD11b^+^CD163^+^ cells. We also verified by flow cytometry that the CD11b^+^CD163^−^ cell population was enriched in CD45^dim^CD11b^+^ microglia (92.9 ± 2.4%, *n* = 2). We then performed additional cell sorting experiments for RNA extraction and gene expression studies (Fig. 2a). By qRT-PCR we detected *Cd163* mRNA expression in the sorted CD163^+^ population but not in the sorted microglia, as expected, whereas the expression of *Aif1* (Iba-1) was higher in microglia than in the CD163^+^ cells, and *Siglec1* (CD169) expression was detected in CD163^+^ cells but not in microglia (Fig. 2b).

**Fig. 3:**
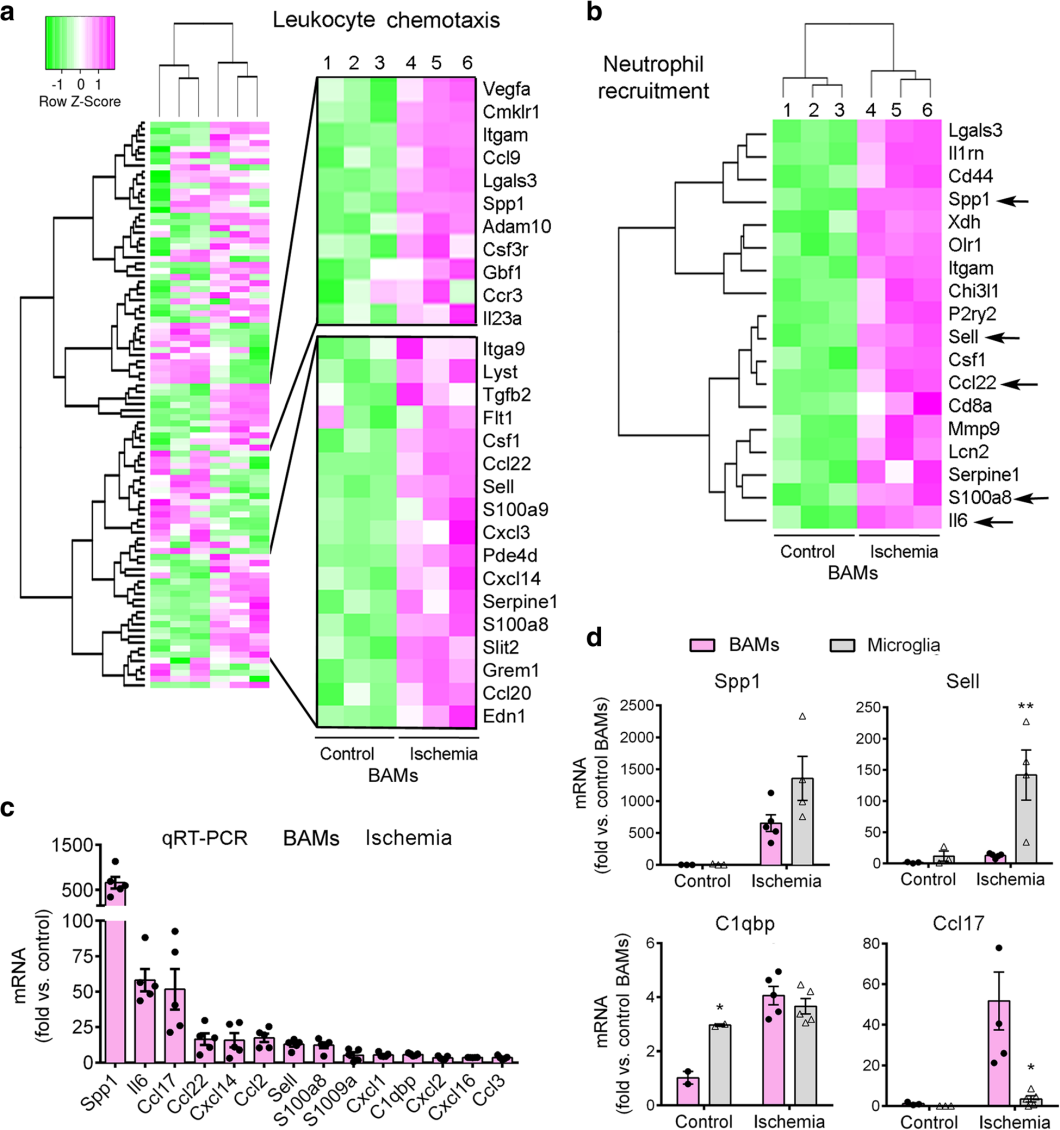
CD163^+^ BAMs upregulate the expression of leukocyte chemoattractants after ischemia. **a**) Heatmap showing the profile expression of upregulated (pink) and downregulated (green) genes in the GO term *Leukocyte chemotaxis*. A set of genes upregulated after ischemia is highlighted. **b**) Heatmap of genes of the IPA category *Recruitment of neutrophils*. Some of these genes (arrows) were later analyzed by qRT-PCR in RNA of cells sorted from a different set of rats. **c**) In a different group of controls (*n* = 3) and ischemic rats (*n* = 5) (16 h of reperfusion), we sorted CD163^+^ BAMs, extracted RNA and carried out qRT-PCR to validate up-regulation of some of the genes previously identified in the microarray. Values are expressed as fold vs. the mean value of control CD163^+^ cells and are shown as the mean±SD. **d**) Gene expression as assessed by qRT-PCR in sorted CD163^+^ BAMs and sorted microglia (*n* = 3 controls and 5 ischemic rats per each cell type). All values are normalized versus the mean value of controls and are represented as the mean±SD. Generally, induction of gene expression after ischemia is similar or lower in BAMs than microglia, with the exception of some genes like *Ccl17* that was induced after ischemia in BAMs but not microglia. Two-way ANOVA followed by the Bonferroni test, *p < 0.05 and **p < 0.01 microglia vs. BAMs

We aimed to investigate whether ischemia changed the gene expression profile of the resident CD163^+^ macrophages. We isolated the CD11b^+^CD163^+^ cells by FACS as above in groups of control non-ischemic rats and rats subjected to ischemia plus 16 h of reperfusion (Additional file 1: Figure S1b). We chose to study this time point after ischemia given that myeloid cell infiltration was still very mild and the majority of CD163^+^ cells were resident macrophages (Fig. 1b,c). We obtained RNA from the sorted cells, and processed it with Affymetrix microarrays. Unsupervised clustering analysis showed that the samples clustered according to the groups (Additional file 1: Figure S2a), and principal component analysis precisely separated the ischemic from the control samples (Additional file 1: Figure S2b). We selected the genes showing statistically significant differences between ischemic and control groups (adjusted *p-*value< 0.01) and filtered for∣logFC∣> 2. According to the above criteria, the expression of 1493 genes was up-regulated whereas the expression of 594 genes was down-regulated in CD163^+^ macrophages after ischemia, as graphically illustrated in the volcano plot (Additional file 1: Figure S2c). The global heat map illustrates differentially expressed genes in these groups (Fig. 2c). The top 25 up-regulated genes (Additional file 1: Table S1) included genes related to the extracellular matrix, such as *Vcan* (versican), *Sdc1* (syndecan), *Fn1* (fibronectin-1), *Spp1* (osteopontin), *Sema3c* (semaphorin 3c), *Mmp7* (matrix metalloproteinase 7, MMP-7), and *Mmp14* (MMP-14) supporting a role of CD163^+^ macrophages in extracellular matrix remodelling. Also, we detected up-regulation of genes involved in nicotinamide adenine dinucleotide metabolism (*Cd38*), and inflammatory and immune responses, like *Tspo, Cd8a,* and *Itgax* (CD11c)*.* The sorted CD163^+^ cells expressed genes recently identified in BAMs of the mouse brain, such as *Mrc1* (CD206), *Mertk*, *Adgre1* (F4.80), *Fcgr3a* (CD16), *C5ar1* (CD88), *Lyve1*, *RT1-ba* (MHC class II antigen), *Cd44, Cd38* [37], and *Tmem119, Csf1r, and Cxcr4* [29]. However, the expression of most of these genes did not significantly change after ischemia, with the exception of increased expression of *Cd38* (Additional file 1: Figure S3A) and *Cd44* (Fig. 3b). Neither control nor ischemic CD163^+^ BAMs showed expression of genes encoding for typical microglial proteins, like *Sall1.* Among the top genes down-regulated after ischemia we found the α-amino-3-hydroxy-5-methyl-4-isoxazolepropionic acid (AMPA) glutamate receptor *Gria2,* genes related to subcellular organization, like *Syne2* and *Fam65b,* and genes of the extracellular matrix, such as *Adam23*, certain growth factor receptors, like *Egfr*, and genes related to immune responses particularly associated to inhibition of leukocyte activation, such as *Siglec5* (Additional file 1: Table S2).

The analysis of biological significance was based on enrichment analysis on different annotation databases to highlight biological categories in which the selected genes appear to be over-represented after brain ischemia. The GO analysis identified over-representation, enriched KEGG entities and clusters for terms related to *acute inflammatory response, cytokine activity, chemokine activity, response to hypoxia, extracellular space, extracellular matrix, metalloproteinases, collagen degradation, blood vessel remodelling, angiogenesis,* and *response to oxidative stress*, amongst others (see Additional file 1: Figure S3 for heat maps of genes in GO-terms representative of the inflammatory response). Down-regulated KEGG pathways included: *ATPase, Ca*^*++*^ *transporting; ATPase, Na*^*+*^*/K*^*+*^ *transporting; Cd7 molecule; KIT ligand; beta-2-adrenergic receptor*; and *epidermal growth factor receptor (Egfr) pathway*. Ingenuity Pathway Analysis (IPA) identified top canonical pathways (Additional file 1: Figure S4A) and top associated disease/functions (Fig. 2d). Amongst the highly up-regulated pathways, *hypoxia inducible factor-1 (HIF-1) signalling* highlighted the response to ischemic conditions (Additional file 1: Figure S4), illustrated by up-regulation of *Hif1a* and vascular endothelial growth factor-A (*Vegfa)* genes (Fig. 3a, Additional file 1: Figure S3). The term *lipopolysaccharide (LPS)* was amongst the top upstream regulators, suggesting acquisition of pro-inflammatory features reminiscent of the response to LPS. However, ischemia-induced CD163^+^ macrophage activation showed a distinctive metabolic and inflammatory signature. For instance, regarding arginine metabolism [38], inducible nitric oxide synthase (iNOS*, Nos2*) was not overrepresented whereas arginase-1 (*Arg1*) was one of the top up-regulated genes (Additional file 1: Table S1). This effect is expected to suppress excessive nitric oxide production and promote L-arginine consumption impairing lymphocyte activation in response to antigen [3, 8].

### Ischemia induces the expression of leukocyte chemoattractants in CD163^+^ BAMs

The above gene pathway analyses in CD163^+^ macrophages highlighted the response to wounding, inflammation, and chemotaxis after ischemia. A heatmap representation is shown for the GO term *Leukocyte chemotaxis* where several genes up-regulated by ischemia are detailed (Fig. 3a). The IPA analysis identified over-representation of the term *Neutrophil recruitment* (Fig. 3b). The analyses showed up-regulation of genes such as *Spp1*, interleukin-6 (*Il6*), C-X-C motif chemokine ligands 14 (*Cxcl14*), *S1008a*, and L-selectin (*Sell*), amongst others. Ischemia also up-regulated genes encoding for chemokines involved in recruiting T helper type 2 (Th2) lymphocytes, like *Ccl22* and *Ccl17* [56] (Fig. 3, Additional file 1: Table S1). For validation purposes, some of the up-regulated genes related to leukocyte chemotaxis were studied by qRT-PCR using RNA of CD163^+^ macrophages sorted from different groups of control and ischemic rats 16 h post-ischemia (Fig. 3c). We compared the expression of these genes in FACS-sorted CD11b^+^CD163^+^ cells versus sorted CD11b^+^CD163^−^ cells enriched in microglia (Additional file 1: Figure S1). CD163^+^ macrophages seemed to work in consonance with microglia to promote leukocyte recruitment to the ischemic brain since most of the genes that we studied by qRT-PCR showed similar (e.g *Spp1, C1qbp*) or weaker (e.g. *Sell*) expression in CD163^+^ macrophages compared to microglia. However, ischemia up-regulated certain genes, such as *Ccl17,* preferentially in CD163^+^ macrophages (Fig. 3d). Altogether, these results suggested the possibility that CD163^+^ macrophages participated in the recruitment of leukocytes after brain ischemia.

### Selective depletion of BAMs but not microglia in control and ischemic brain

**Fig. 4:**
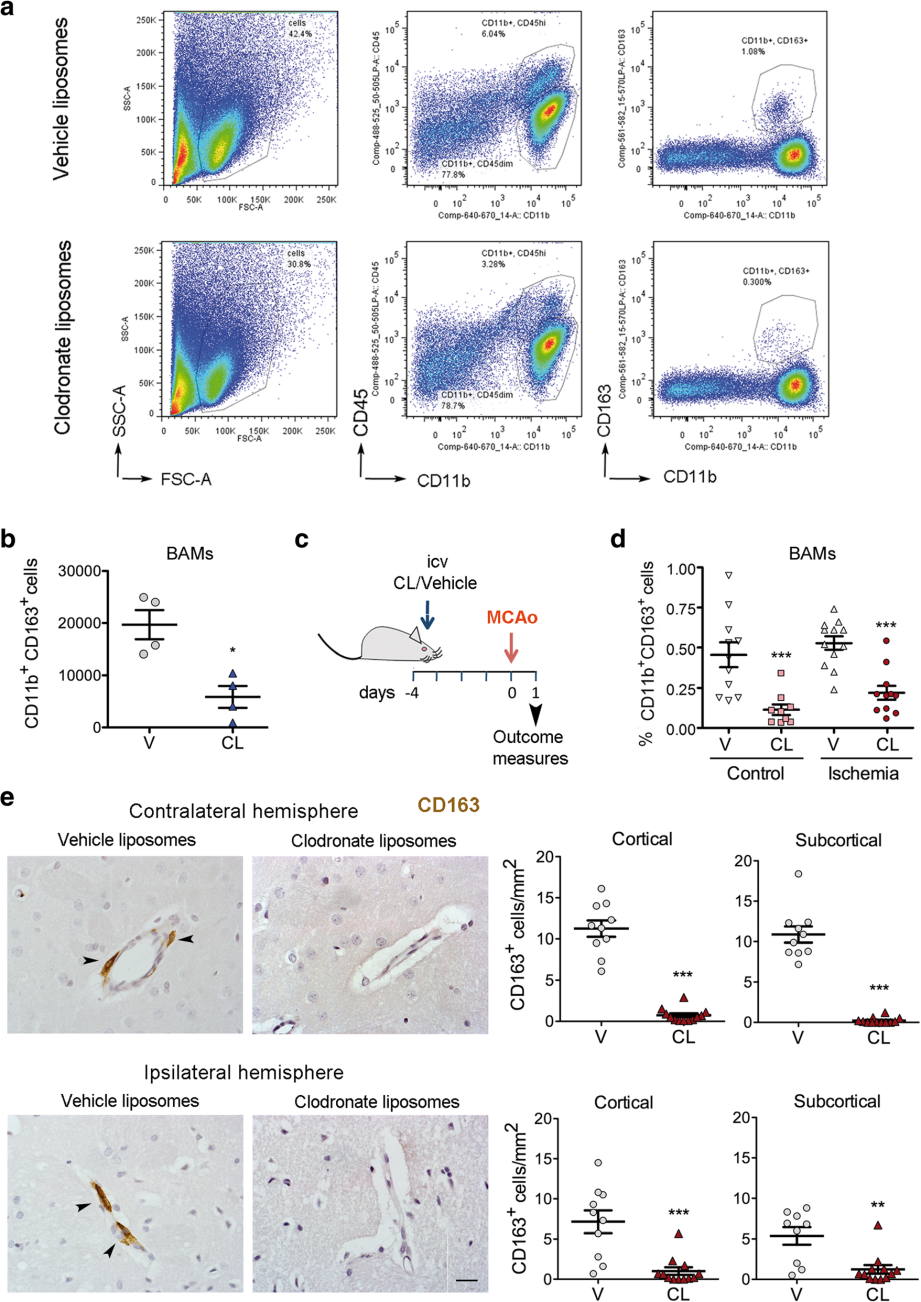
Treatment with clodronate liposomes depleted BAMs in control and ischemic rats. **a**) Flow cytometry plots of CD11b^+^CD163^+^ cells in the brain of control rats treated i.c.v. with either vehicle liposomes (V) or clodronate liposomes (CL) 5 days before the analysis. **b**) CL reduced the numbers of brain CD11b^+^CD163^+^ cells (*p < 0.05, *n* = 4 per group). **c**) Rats received CL or V in the left ventricle 4 days before occlusion of the right MCA and the brain was analyzed by flow cytometry 1 day after ischemia. **d**) Flow cytometry analysis of CD11b^+^ CD163^+^ cells of the ischemic hemisphere (*n* = 12 V; *n* = 11 CL) and the corresponding hemisphere of control rats (*n* = 11 V; *n* = 9 CL) and shows reduction after treatment with CL versus V in both groups. *** *p* < 0.001, two-way ANOVA by treatment and condition. **e**) Immunohistochemistry and cell counting in brain shows reduced numbers of CD163^+^ cells in the contralateral and ipsilateral (infarcted core) cortical and subcortical regions of the CL group (n = 12) compared to the V group (*n* = 10). *** *p* < 0.001, ** *p* < 0.01. Scale bar: 20 μm

To obtain functional information on the role of BAMs in the acute phase of stroke, we carried out loss-of-function experiments by intracerebroventricular (i.c.v.) administration of liposomes containing clodronate [14, 23, 42, 43]. Macrophages scavenge the liposomes and the intracellular accumulation of the toxin clodronate induces macrophage cell death [46, 55]. Liposomes containing either clodronate or vehicle (PBS) were administered in the left ventricle and the brain was analyzed 5 days later. Flow cytometry studies of the brain cells showed that clodronate liposomes induced a strong reduction (65–70%) of the CD163^+^ population in both hemispheres (Fig. 4a,b). We then checked that CD163^+^ cell depletion was maintained after brain ischemia. We occluded the right MCA four days after administration of clodronate or vehicle liposomes to the left ventricle and studied the brain tissue 24 h post-ischemia (Fig. 4c). CD163^+^ cells were also reduced in the ischemic tissue after clodronate treatment, as assessed by flow cytometry (Fig. 4d). Immunohistochemistry (Fig. 4e) showed depletion of CD163^+^ macrophages located in the perivascular spaces and on the glia limitans under the pia matter. Of note, we verified that i.c.v. clodronate liposome treatment did not significantly affect the number of parenchymal microglia compared to the vehicle group in the control and ischemic brain tissue, as shown by immunohistochemistry (Iba1^+^) (Additional file 1: Figure S5a), and flow cytometry (CD45^dim^CD11b^+^) (Additional file 1: Figure S5b). In both hemispheres of the clodronate group we observed the presence of CD163^−^ Iba1^+^ perivascular cells (Fig. 4e, Additional file 1: Figure S5b) that might correspond to peripheral myeloid cells. The effect of i.c.v. administration of clodronate was local and it did not modify blood cell counts (Additional file 1: Figure S6).

### BAM depletion ameliorated the stroke-induced neurological dysfunction

**Fig. 5:**
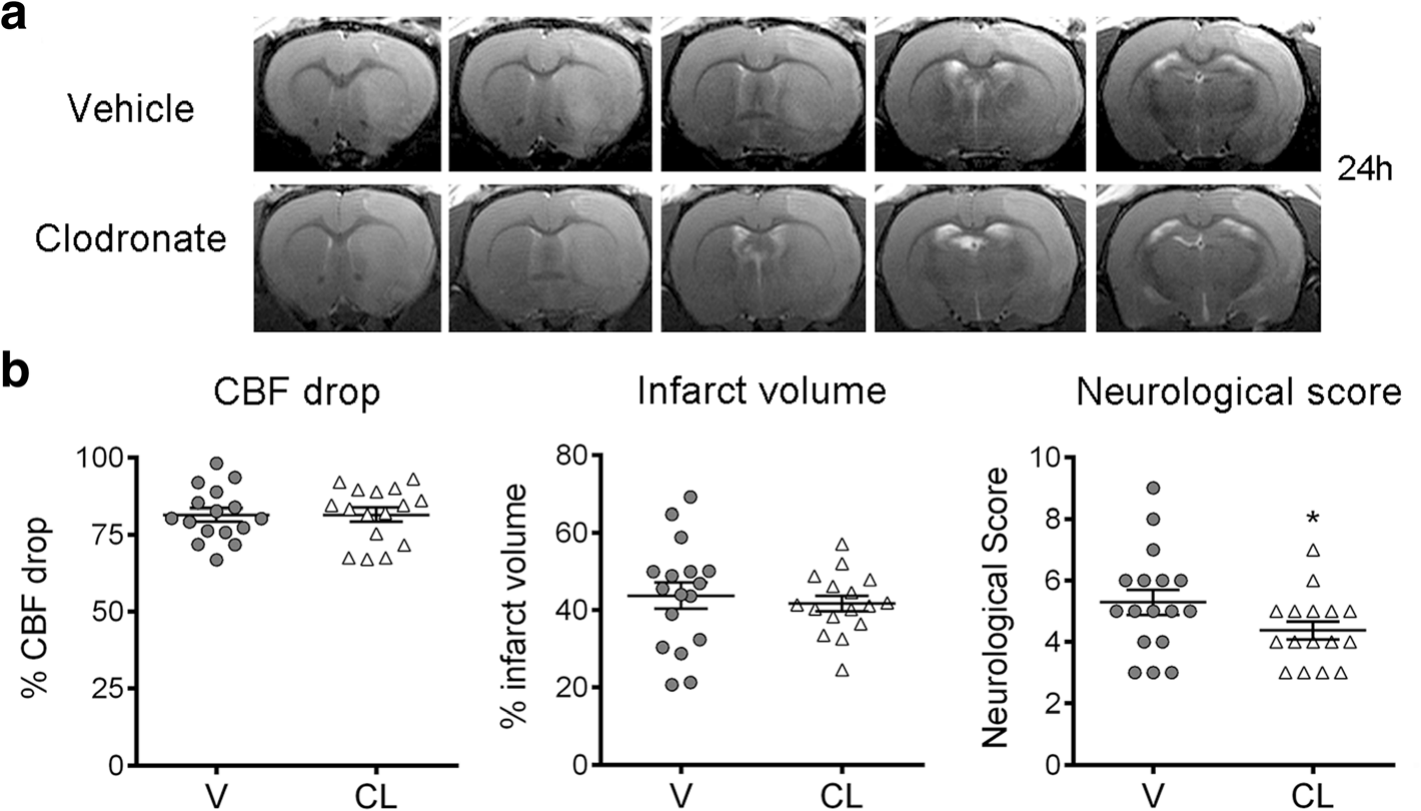
Depletion of BAMs does not reduce infarct volume but attenuates the neurological deficit. **a** T2w MRI 24 h post-ischemia of representative rats per treatment group, i.e. clodronate (CL, *n* = 16) or vehicle (V, *n* = 17) liposomes. **b** Ischemia induced a similar drop of cortical perfusion (CBF) during MCAo, as assessed by laser Doppler, in both groups. The neurological score shows a small but significant improvement in the CL group. (Mann-Whitney test, * *p* = 0.046) There are no differences between groups in total infarct volume (**b**), as well as the volume of infarcted cortical and subcortical regions (not shown), as assessed by T2w MRI. Treatment, ischemia, neurological evaluation, MRI, and infarct volume measures were carried out in a blinded fashion

To find out whether BAM depletion affected the extent of brain damage we measured the size of the brain lesion and assessed the neurological function 24 h post-ischemia in rats treated with clodronate or vehicle liposomes, as above. One-hour ischemia induced large brain lesions and BAM depletion did not cause a major effect in the volume of infarction (Fig. 5). However, rats depleted of BAMs showed a mild but significant improvement of the neurological score (Fig. 5), suggesting that BAM activity has negative consequences for the neurological status in the acute phase of stroke.

### BAM depletion alters leukocyte recruitment after brain ischemia

**Fig. 6:**
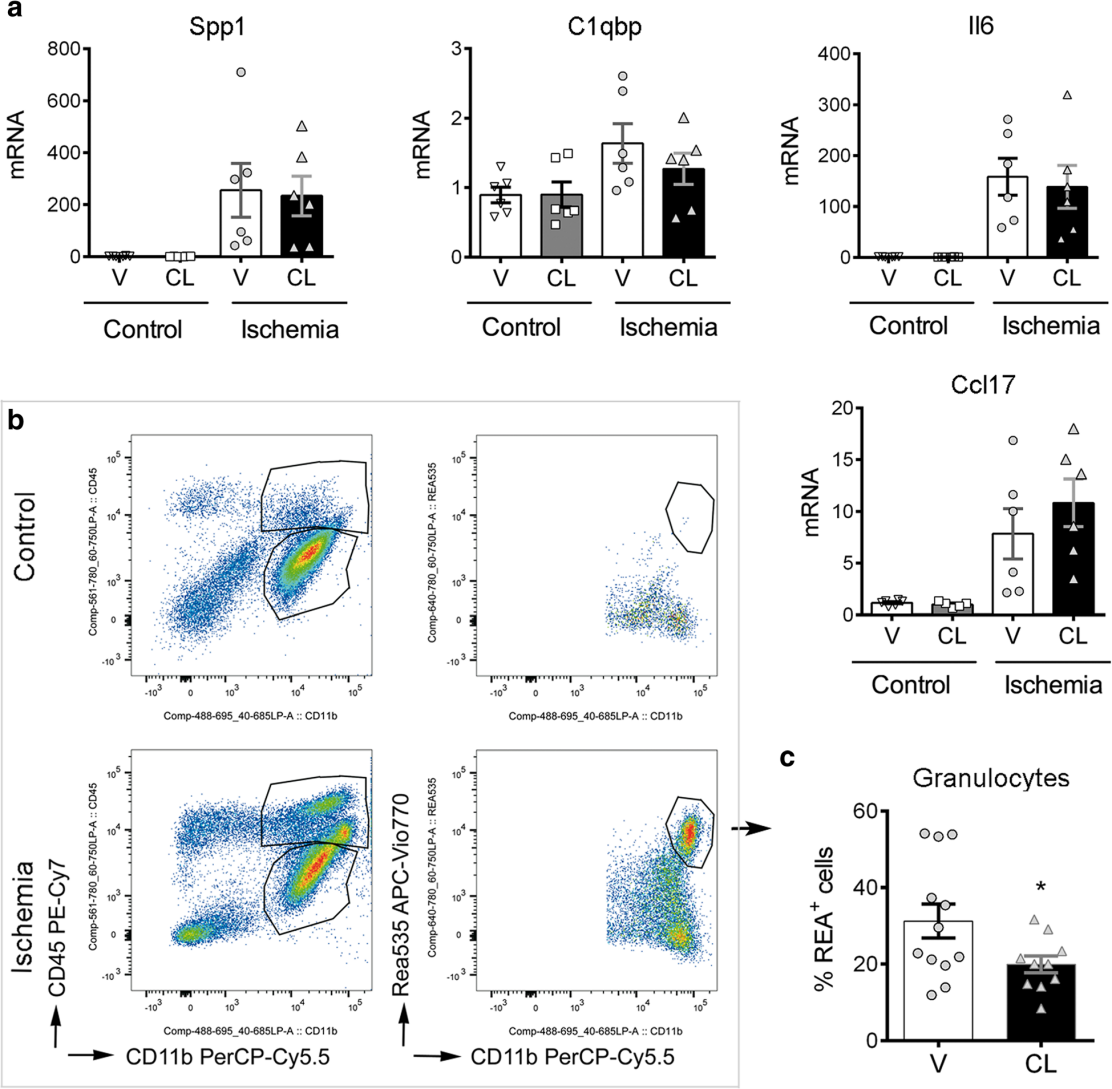
Effects of BAM depletion on leukocyte infiltration after brain ischemia. Rats received clodronate (CL) or vehicle (V) liposomes in the left ventricle 4 days before occlusion of the right middle cerebral artery and the brain was analyzed 1 day after ischemia. **a**) mRNA was extracted from the contralateral (control) and ipsilateral (ischemic) brain hemispheres of rats treated with V (*n* = 7) or CL (*n* = 6) for qRT-PCR analysis. Values are expressed as fold versus the mean value in the control V group. CL does not reduce leukocyte chemoattractants that were upregulated by ischemia in CD163^+^ macrophages (see Fig. 3), such as *Spp1, C1qbp, Il6,* and *Ccl17*. **b**) Flow cytometry analysis of the brain hemisphere of control rats (*n* = 11 vehicle, V; *n* = 9 CL) and ischemic rats (*n* = 12 V; *n* = 11 CL) illustrates leukocyte infiltration in the ischemic group. **c**) Depletion of CD163^+^ macrophages (CL group) reduces the percentage of REA535^+^ granulocytes in the infiltrating myeloid cell population (CD45^hi^CD11b^+^) (*p = 0.039)

**Fig. 7:**
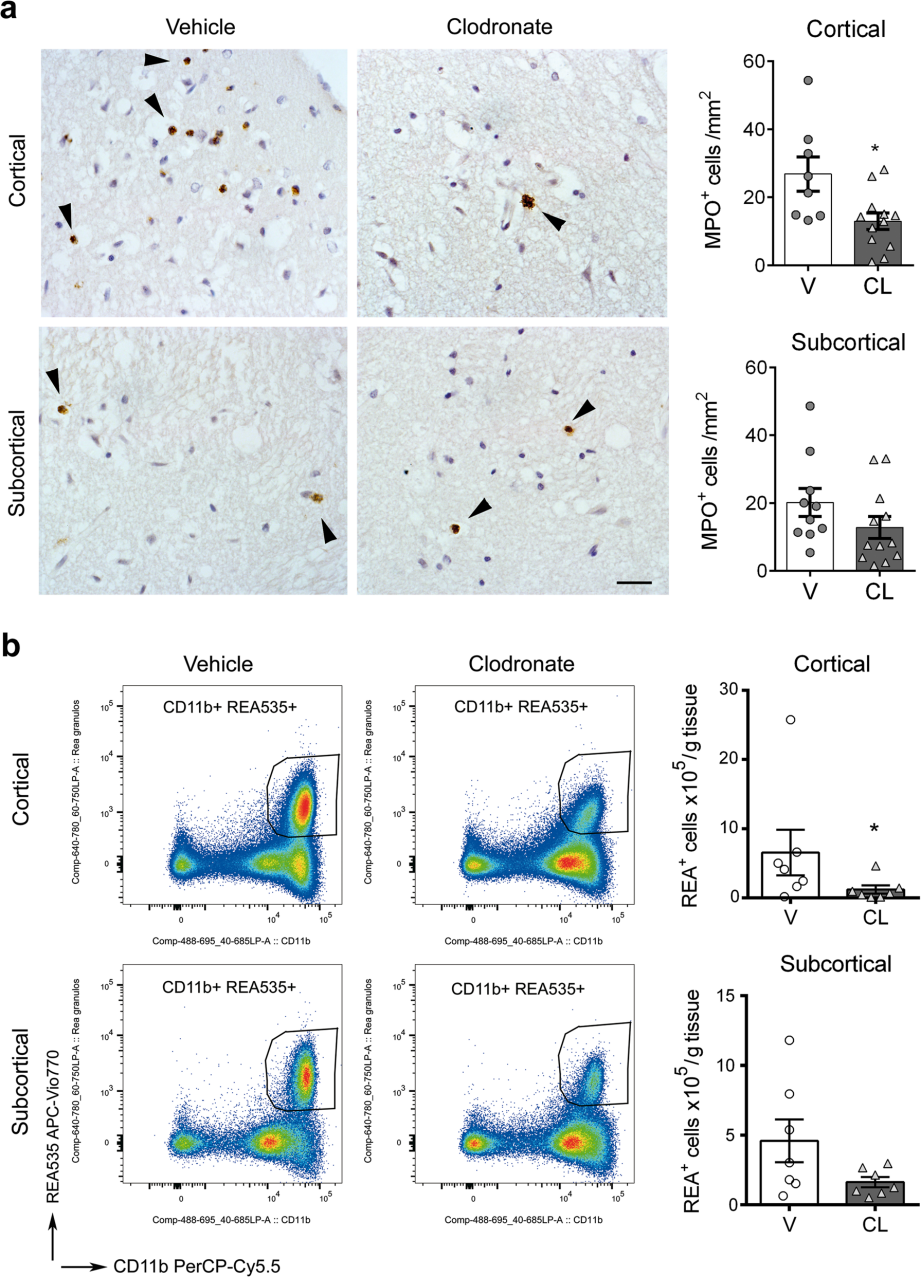
Depletion of BAMs reduces granulocyte infiltration in the ischemic cortex. Rats received clodronate (CL) or vehicle (V) liposomes in the left ventricle 4 days before occlusion of the right middle cerebral artery and the brain was analyzed 1 day after ischemia. **a**) Myeloperoxidase (MPO) immunohistochemistry (brown, arrows) of the core of infarction in paraffin sections (counterstained with hematoxylin in blue) of ischemic rats treated with V or CL. Scale bar: 20 μm. Blind cell counting showed that CL (*n* = 12) reduces the numbers of MPO^+^ cells in the ischemic cortex compared to the V (*n* = 8) (*p = 0.05), but differences in subcortical regions were not statistically significant (CL *n* = 12, V *n* = 10, *p* = 0.12). **b**) An independent group of rats treated with V or CL was studied by flow cytometry 24 h postischemia after dissection of cortical and subcortical brain regions. The numbers of REA535^+^ granulocytes was determined. Values are expressed as number of cells per gram of brain tissue. The number of REA535^+^ cells decreased in both regions after BAM depletion. However, differences between treatment groups were statistically significant in cortical regions (*p = 0.038), whereas only a trend was observed in subcortical regions (*p* = 0.085)

We then investigated by qRT-PCR whether depletion of BAMs altered the RNA expression of cytokines and chemokines in the brain tissue 24 h after ischemia. The brain of rats treated with clodronate liposomes did not show a significant reduction in the mRNA expression of the leukocyte chemoattractants and chemokines that we examined (Fig. 6a). This result is compatible with ischemia-induced expression of these genes in other cells in addition to CD163^+^ macrophages, including microglia and potentially astrocytes or other neural cells. In spite of this, depletion of CD163^+^ cells reduced the percentage of infiltrating REA535^+^ granulocytes in the ischemic hemisphere, as determined by flow cytometry (Fig. 6b,c) whereas the numbers of other leukocytes were not affected (Additional file 1: Figure S7). To validate this finding, we carried out immunohistochemistry with anti-myeloperoxidase antibodies in an independent group of rats. Blind cell counting showed less infiltrating granulocytes after BAM depletion, particularly in cortical regions (Fig. 7a). To further investigate possible regional differences we carried out a second flow cytometry study in an independent group of ischemic rats where we dissected out cortical and subcortical regions and analysed them separately. BAM depletion reduced REA535^+^ granulocyte numbers in the ischemic cortex, whereas subcortical regions only showed a non-significant trend (Fig. 7b). In contrast to the effect on granulocyte recruitment, BAM depletion did not significantly modify the numbers of T cells, NK cells, or myeloid mononuclear cells infiltrating the ischemic tissue (Additional file 1: Figure S8).

### BAMs increase cortical vascular permeability 24 h after ischemia

**Fig. 8:**
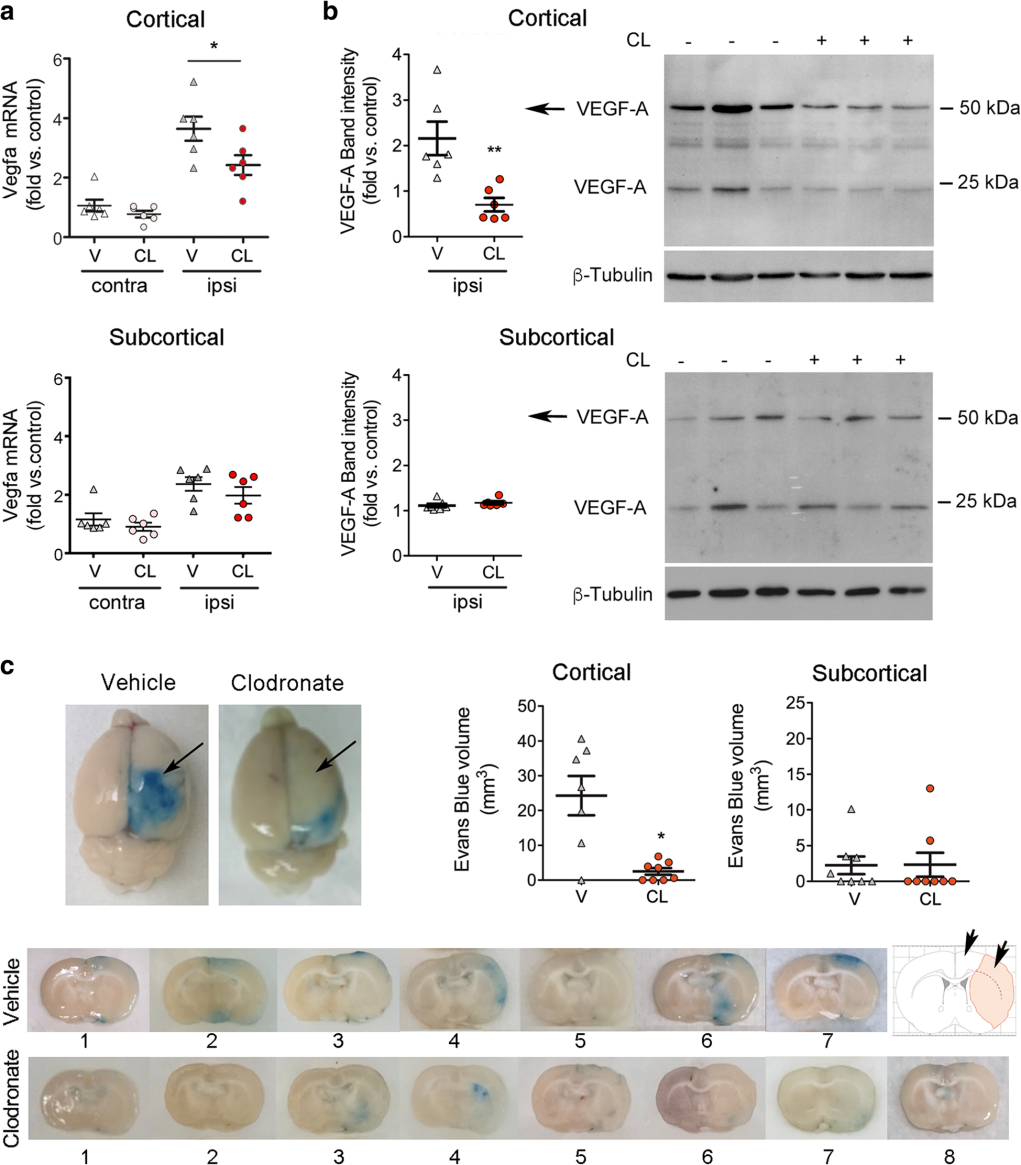
Depletion of BAMs attenuates ischemia-induced pial and cortical vascular permeability. BAMs were depleted by i.c.v. administration of clodronate liposomes (CL) 4 days prior to ischemia. For treatment control, rats received vehicle liposomes (V). **a**) *Vegfa* mRNA was studied in cortical and subcortical regions of the ipsilateral (ipsi, ischemic) and the contralateral (contra) brain hemispheres. Ischemia-induced expression of *Vegfa* mRNA at 24 h is attenuated in the cortex of the CL rats versus the V rats (*n* = 6 per group) (Mann Whitney test, *p* = 0.041). **b**) Western blotting of cortical and subcortical brain tissue (ipsilateral) 24 h post-ischemia shows the VEGF_164_ isoform of VEGF-A detected as two bands corresponding to the homodimeric and monomeric forms at approximately 54 kDa and 24 kDa. Quantification of signal intensity of the 54-kDa band shows reduced expression in the cortex of rats receiving CL vs. rats receiving V (*n* = 6 per group) (Mann-Whitney test, **p = 0.002). **c**) Ischemia increases the permeability of pial vessels (arrows), as assessed by Evans blue extravasation 24 h post-ischemia, and BAM depletion attenuates the effect. Images of one representative coronal brain section per rat of each treatment group show Evans blue extravasation in the ipsilateral hemisphere. Rats receiving clodronate show negligible Evans blue in the cortex (arrows in the schematic representation at the right hand side). The volume of tissue showing Evans blue extravasation is reduced in the cortex, but not in subcortical zones, of the CL group (*n* = 8) versus the V group (*n* = 7) (Mann-Whitney test, *p = 0.014)

The microarray analysis of sorted CD163^+^ macrophages identified over-representation of genes involved in the post-ischemic response to hypoxia, such as the HIF-target gene *Vegfa* (Fig. 3a, Additional file 1: Figure S3a). BAM depletion reduced the expression of *Vegfa* mRNA in the cerebral cortex 24 h post-ischemia, whereas this effect was milder in subcortical regions (Fig. 8a). Ischemia induced the expression of VEGF_164_ protein (Fig. 8b), a VEGF-A isoform (similar to human VEGF_165_) that is secreted and has the capacity to bind to the extracellular matrix [45]. In agreement with the mRNA results, BAM depletion reduced the cortical expression of VEGF_164_ (Fig. 8b).

Given that hypoxia-induced VEGF in the brain produces acute vascular leakage [48], we studied whether BAM depletion affected vascular permeability. Notably, ischemia-induced Evans blue extravasation from the ipsilateral pial vessels and cortex was minimal in rats depleted of BAMs (Fig. 8c), which showed a reduced volume of tissue with Evans blue extravasation in the cortex but not in subcortical regions. These results suggest that depletion of BAMs prevented ischemia-induced leakage of pial and cortical vessels.

**Fig. 9:**
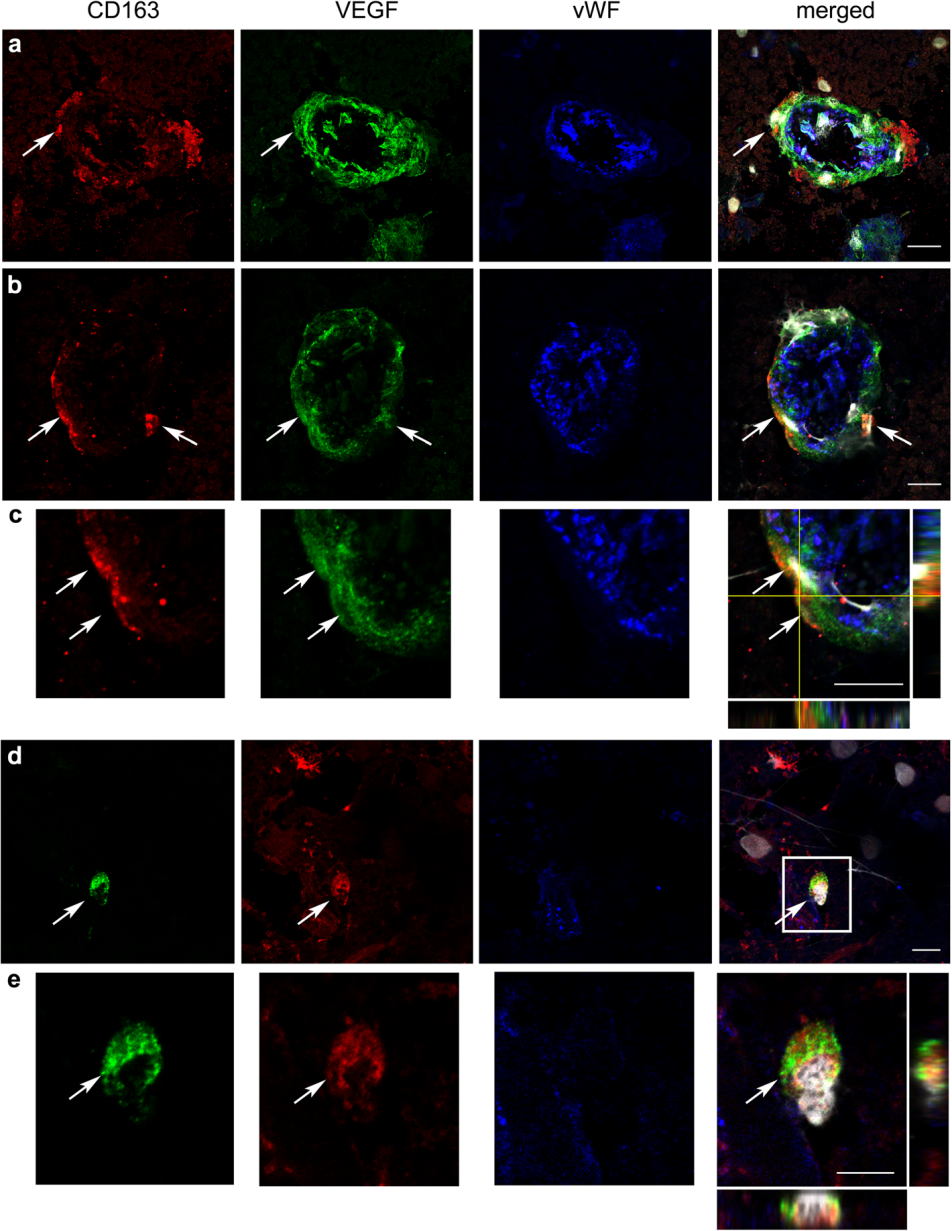
CD163+ macrophages and neighboring vessels express VEGF in human ischemic stroke. VEGF around the vessels and in surrounding CD163^+^ macrophages in human ischemic stroke. Immunofluorescence staining of post-mortem brain tissue of patients deceased 24 h after ischemic stroke (*n* = 3). Confocal microscope images show CD163^+^ macrophages (red in **a**-**c**, green in **d**-**e**), vascular endothelium (von Willebrand factor, vWF; blue), and cell nuclei (white) that are stained with DAPI. The presence of VEGF immunoreactivity (green in **a**-**c**, red in **d**-**e**) is observed surrounding the endothelial layer of blood vessels and in CD163^+^ macrophages. Images in **c** and **e** are magnifications of the indicated part of **b** and **d**, respectively. Orthogonal projections show co-localization of VEGF and CD163 staining in some cells. The arrows point to CD163^+^ perivascular macrophages positive for VEGF. Scale bar: 10 μm

We then investigated whether these findings might be relevant to acute ischemic stroke in humans. To this end, we studied CD163^+^ macrophages in post-mortem brain tissue of three patients with fatal ischemic stroke deceased 24 h after symptom onset. VEGF immunoreaction was detected surrounding blood vessels and we found some CD163^+^ perivascular macrophages positive for VEGF (Fig. 9). We observed that VEGF immunoreactivity was more prominent in the two patients that did not receive any revascularization therapy than in the patient that received mechanical thrombectomy. This effect is likely attributable to persistent ischemic conditions in the former. The described perivascular VEGF immunoreaction was mainly found at the periphery of the infarcted core. In the infarcted core, we also observed VEGF immunoreactivity in parenchymal CD163^−^ cells with a pattern of expression that varied across the tissue and between patients. Altogether, these results are compatible with the possibility that human CD163^+^ perivascular macrophages secreted VEGF in acute ischemic stroke.

## Discussion

BAMs are players at the interface between the nervous system and the immune system [6, 12, 27, 29, 34, 37, 53]. However, the precise functions of these cells under pathological conditions remain largely unknown. This study provides evidence for a role of BAMs increasing vascular permeability, facilitating granulocyte recruitment, and contributing to neurological dysfunction in the acute phase of ischemic stroke. BAMs express typical myeloid cell markers like microglia but their differential phenotypic features were less known until recently published studies provided an extensive description of the phenotype of BAMs in the mouse brain [29, 37]. We verified that rat CD163^+^ BAMs expressed genes encoding for proteins identified in these latter works. However*,* the expression of most of these BAM genes did not significantly change 16 h post-ischemia versus controls, with the exception of increased expression of *Cd38* and *Cd44*. Although CD44 expression was previously considered as a marker of brain infiltrating cells [29], we detected the expression of *Cd44* in CD163^+^ BAMs obtained from the control rat brain, in agreement with the finding of CD44^+^ BAMs in the control mouse brain [37]. Furthermore, the expression of *Cd44* increased after ischemia, as described for CD44 expression in mouse BAMs in EAE [37]. In addition, we found that rat CD163^+^ BAMs express *Siglec1* (CD169) and low levels of *Aif1* (Iba-1), in contrast to microglial cells that do not express *Siglec1* and express comparatively higher levels of *Aif1*.

Brain ischemia causes necrotic neuronal cell death with release of danger signals that activate the microglia [26], but the response of BAMs is currently unknown. We found that these cells respond to ischemic conditions by showing changes in the gene expression profile enabling adaptation to hypoxia and acquisition of novel cellular functions involved in extracellular matrix-vascular interactions and inflammatory responses. BAMs upregulated the expression of genes regulating neutrophil chemotaxis as part of the reprogramming process induced by ischemia. The anatomic location of these cells confers them the capacity to participate in the communication network between the brain environment and the vasculature. Brain ischemia induces neutrophil extravasation from leptomeningeal vessels and neutrophil accumulation in perivascular spaces of the affected brain region [9, 40]. Our study suggests that CD163^+^ macrophages attract granulocytes to the leptomeningeal and perivascular spaces in response to brain ischemia. These findings are in consonance with a study showing that perivascular macrophage depletion reduced brain neutrophil infiltration in a model of vesicular stomatitis viral infection [51]. Furthermore, perivascular macrophages mediate neutrophil recruitment during bacterial skin infection [1]. Other works reported the involvement of mononuclear cells in neutrophil attraction. For instance, specific subsets of monocytes are key regulators of neutrophil extravasation in the lung [30].

Functional analysis of genes over-represented in CD163^+^ BAMs after brain ischemia versus controls highlighted the activation of neovascularization processes and the HIF-1 pathway. This finding is consistent with the observed up-regulation of the HIF-1α target gene *Vegfa,* a potent modulator of angiogenesis [2, 32] and regulator of the cellular redox status [11]. We observed the expression of VEGF in the post-mortem human brain tissue of stroke patients deceased 24 h after stroke onset, and detected VEGF surrounding some brain vessels and CD163^+^ perivascular macrophages, suggesting that the findings in rats might be valid in acute ischemic stroke patients. Acute production of VEGF is associated with increases in vascular permeability [48]. In our study, BAM depletion in the rats decreased the ischemia-induced expression of *Vegfa* mRNA and the secreted protein isoform, VEGF_164_, in the cerebral cortex. Furthermore, BAM depletion reduced Evans blue extravasation from pial and cortical vessels, whereas these effects were less marked in subcortical regions. We argue that the greater cortical effect might be due to the activity of subpial macrophages increasing the permeability of pial vessels in the acute phase of stroke. While we were preparing this manuscript, a study reported pathogenic actions of CD163^+^ macrophages in atherosclerosis where these cells showed activation of HIF1α and VEGF expression and increased vascular permeability and inflammatory cell recruitment [20]. Interestingly, perivascular macrophages are involved in maintaining vascular barrier function in the periphery under physiological conditions [21, 24]. Here we show that an ischemic stroke challenge changes the homeostatic function of CD163^+^ macrophages by inducing cellular activation of the HIF pathway and generation of VEGF and inflammatory mediators. This effect in the very acute phase of stroke does not exclude putative vasculoprotective effects of perivascular macrophages in chronic phases after stroke.

Our study did not find any differences in infarct volume 24 h post-ischemia in rats depleted of BAMs, in spite that they showed reduced granulocytes in the ischemic brain. Previous studies reported reduced infarct volume after ischemia/reperfusion in rats depleted of neutrophils [35]. However, the effects were not entirely reproduced in a model of severe ischemia/reperfusion in rats [22], or in a model of ischemia/reperfusion plus thrombolytic treatment in spontaneous hypertensive rats where only one out of two different neutrophil depletion strategies reduced the lesion size [16]. Also, neutrophil depletion reduced infarct size in hyperlipidemic ApoE^−/−^ mice but not in normolipidemic wild-type mice [25]. Therefore, it was unlikely that the moderate reduction of granulocyte recruitment induced by BAM depletion in our model of severe ischemia/reperfusion was sufficient to cause major differences in the size of the severe brain lesion induced by our ischemia model. Nevertheless, we found some improvement of the neurological function in the absence of BAMs that was attributable to attenuated granulocyte recruitment and preservation of pial and cortical vascular integrity after ischemia.

A limitation of our study is that it only addressed the function of BAMs in the acute phase of stroke. We restricted our study to the first hours after stroke to avoid the putative confounding effect of massive macrophage infiltration that takes place in the days that follow stroke [5, 17, 47, 49, 52]. Moreover, our BAM depleting strategy is transient and repopulation of these cells by peripheral macrophages could follow at later time points. Long-term studies using different experimental approaches are required to understand the consequences of BAM activity for the evolution of the brain lesion and the repair processes, particularly regarding extracellular matrix and vascular remodelling. Another limitation is that we could not separate perivascular from subpial macrophages since all of them express CD163 in the rat brain. Depleting the CD163^+^ macrophage population had more effects in cortical rather than in subcortical regions after ischemia. We suspect that subpial macrophages may play a role by producing VEGF and regulating vascular permeability and extravasation of granulocytes from pial vessels. These effects could exacerbate the brain lesion by impairing the integrity of the collateral circulation [50]. Finally, we conducted our experimental study in young male rats, while stroke mainly affects old people of both sexes. Studies in aged animals and females are required to find our whether BAMs show a similar response in these conditions.

## Conclusions

In summary, this study provides evidence for a role of BAMs in granulocyte chemoattraction and vascular permeability following acute ischemic stroke. The study also suggests that targeting these potentially negative responses of brain resident macrophage might help to preserve cortical blood supply and improve the neurological function in the acute phase of cerebral ischemia/reperfusion.

## Additional file

Additional file 1: Extended methods, additional tables and figures.ᅟ(DOCX 6178 kb)

## Abbreviations

Aβ: Amyloid-β
AMPA: α-amino-3-hydroxy-5-methyl-4-isoxazolepropionic acid
BAMs: CNS border-associated macrophages
CNS: Central nervous system
Cxcl: C-X-C motif chemokine ligand
EAE: Experimental autoimmune encephalomyelitis
Egfr: Epidermal growth factor
FACS: Fluorescence activated cell sorting
HIF-1: Hypoxia inducible factor-1
IL: Interleukin
iNOS: Inducible nictric oxide synthase
IPA: Ingenuity Pathway Analysis
LPS: Lipopolysaccharide
MCA: Middle cerebral artery
MCAo: MCA occlusion
PBS: Phosphate-buffered saline
Th2: T helper type 2
VEGF: Vascular endothelial growth factor
