# Supplementary material for: CNS-border associated macrophages respond to acute ischemic stroke attracting granulocytes and promoting vascular leakage
Source: Acta Neuropathol Commun. 2018 Aug 9;6:76. doi: 10.1186/s40478-018-0581-6 (PMC6083589; doi:10.1186/s40478-018-0581-6)
Supplement: Supplementary file 1 — Extended methods, additional tables and figures.ᅟ(DOCX 6178 kb) [file 40478_2018_581_MOESM1_ESM.docx]

**SUPPLEMENTAL INFORMATION**

**CNS-border associated macrophages respond to acute ischemic stroke attracting granulocytes and promoting vascular leakage**

Jordi Pedragosa^1,2^, Angélica Salas-Perdomo^1^, Mattia Gallizioli^1,2^, Roger Cugota^1^, Francesc Miró-Mur^2,3^, Ferran Briansó^4,5^, Carles Justicia^1,2^, Fernando Pérez-Asensio^1^, Leonardo Marquez-Kisinousky^1^, Xabier Urra^2,6^, Anna Gieryng^7^, Bozena Kaminska^7^, Angel Chamorro^2,6^, Anna M. Planas^1,2*^

^1^ Department of Brain Ischemia and Neurodegeneration, Institut d’Investigacions Biomèdiques de Barcelona (IIBB)-Consejo Superior de Investigaciones Científicas (CSIC).

^2^ Àrea de Neurociències, Institut d’Investigacions Biomèdiques August Pi i Sunyer (IDIBAPS), Barcelona, Spain.

^3^ Fundació Clínic, Hospital Clínic de Barcelona, Barcelona, Spain.

^4^ Statistics and Bioinformatics Unit (UEB), Vall d'Hebron Research Institute (VHIR), Barcelona, Spain.

^5^ Department of Genetics, Microbiology and Statistics, Universitat de Barcelona, Barcelona, Spain

^6^ Functional Unit of Cerebrovascular Diseases, Hospital Clínic, Barcelona, Spain

^7^ Laboratory of Molecular Neurobiology, Neurobiology Center, Nencki Institute of Experimental Biology, Warsaw, Poland

**EXTENDED METHODS**

***Animals***

Adult male Sprague-Dawley rats (280-320 g body weight) were obtained from Charles River (Lyon, France). Animals were maintained in a 12h light/dark cycle, under controlled environmental conditions, and with free access to food and water.

***Brain Ischemia in rats***

Focal brain ischemia was induced in rats by 1 hour intraluminal occlusion of the right middle cerebral artery (MCAo) with reperfusion, as reported [6,10], with some modifications. Briefly, rats received analgesic (Buprenorphine, 0.05 mg/Kg) and were anaesthetised with 5 % isoflurane in a mixture of 30% O_2_ and 70% N_2_O. Anaesthesia was maintained with 2 % isoflurane in the same mixture by the aid of a facial mask. The fronto-parietal cortical perfusion was measured with a laser–Doppler flowmeter (PF4001 Master, Perimed). Body temperature was maintained at 37.5°C during surgery with a heating blanket connected to a rectal probe. The common carotid arteries were occluded with vascular clips and the right MCA was occluded with the intraluminal technique (Doccol filament #403912PK10Re). After 50 min, the clip of the contralateral CCA was removed, and 10 min later, the MCA filament and the ipsilateral CCA clip were removed. After suturing, the rats were maintained in a cage on a thermic pad for 90 min to allow recovery from the anaesthesia and were killed 16h or 24h later. Rats were excluded from the study if the mean drop in cerebral perfusion during ischemia did not reach at least 65% of the basal value (n=5, 3 vehicle and 2 clodronate). Nine rats died before 24h post-ischemia (3 vehicle and 5 clodronate). A simple neurological test on a nine-point scale (0=no deficit to 9=highest handicap) was performed at 24 hours, as described [6]. In brief, we scored: i) spontaneous activity (moving/exploring=0, moving without exploration=1, no moving or only when pulled by the tail=2); ii) circling to the left (none=0, circling when elevated by the tail and pushed or pulled=1, spontaneous circling=2, circling without displacement=3); iii) resistance to left (contralateral) forepaw stretching (full resistance=0, rats offer some resistance but they allow stretching=1, rats offer no resistance =2), and iv) the parachute reflex (symmetrical=0, asymmetrical=1, contralateral forelimb retracted=2).

***Drug administration***

Rats received an i.c.v. injection of liposomes containing either clodronate (5 µg/µl) or phosphate-buffered saline (PBS) as the vehicle (ClodronateLiposomes.com, Haarlem, The Netherlands) [7]. Rats were anesthetized with isoflurane and were placed in a stereotaxic apparatus. A volume of 30 μl of liposome solution was slowly administered (during 25 min), with a Hamilton syringe connected to a micro-pump, in the left lateral ventricle at the following coordinates in relation to Bregma: 0.9 mm posterior, 1.5 mm lateral, and 3.5 mm dorso-ventral (after initially introducing the needle down to 4.5 mm dorso-ventral and then pulling it back 1mm to start the infusion at 3.5 mm). Treatment was randomly allocated following a generated code list ([www.graphpad.com/quickcalcs/randomize2/](http://www.graphpad.com/quickcalcs/randomize2/)), and it was administered in a blinded fashion by labelling the vials with a code that did not reveal treatment identity. The researchers carrying out administration, ischemia, and outcome measures were blinded to the treatment. Ischemia was induced 4 days later by occluding the right MCA as described.

***MRI evaluation of infarct volume***

The brain was imaged 24h after MCAo with MRI in a 7.0 T horizontal animal scanner (BioSpec, Bruker BioSpin, Ettlingen, Germany) equipped with an actively shielded gradient system (400 mT/m, 12 cm inner diameter), as reported [9]. Brain lesions were evaluated by T2 mapping of coronal slices acquired with a multislice-multi-echo sequence by applying 25 echo times, between 8 to 200 ms, TR = 4000 ms, acquisition time = 17m 4s, slice thickness = 1 mm, number of slices = 18, FOV = 40 x 40 mm, and matrix size = 256 x 256 pixels, resulting in a spatial resolution of 0.156 x 0.156 mm/pixel. Images were reconstructed with Paravision 5.1 software (Bruker Biospin, Etlingen, Germany). Infarct volume was analyzed using ImageJ and was corrected for edema by multiplying the infarct volume by the ratio of the contralateral to the ipsilateral hemisphere volumes. The brain of the animals studied by MRI was used for the first flow cytometry study and for the Evans blue study.

***Evans blue extravasation***

We studied Evans blue extravasation to the brain tissue as reported [6]. In brief, a solution of 2% Evans blue (Sigma-Aldrich) (w/v in saline) was administered i.v. (4 mL/Kg of body weight) 22 hours after ischemia and 2 hours later the animals were anesthetized and perfused through the heart with saline. The brain was sectioned in 2-mm coronal sections and pictures were obtained. Images were analysed with ImageJ software to measure the volume of tissue with Evans blue extravasation. In one slice the ipsilateral and contralateral cortex and striatum were dissected out and kept frozen at -80ºC until further analysis for Western blotting.

***Western blotting***

Brain tissue was homogenized in lysis buffer containing a cocktail of protease inhibitors, sonicated, and centrifuged at 12,000 xg for 10 min at 4ºC. The protein content in the supernatant was determined with the Bradford assay. Proteins were separated by electrophoresis in 12% polyacrilamide gels and were transferred to polyvinylidene fluoride membranes that were incubated overnight at 4ºC with a goat polyclonal antibody against VEGF-A (#AF564, R&D) diluted 1:500, followed by a secondary anti-goat HRP antibody diluted 1:2,000 (Rabbit anti-goat IgG (whole molecule)-HRP conjugate, #A5420, Sigma-Aldrich). As the loading control, we used a mouse monoclonal antibody against β-tubulin (#T4026, Sigma) diluted 1:500,000, followed by a secondary antibody (Goat anti-mouse IgG (H+L)-HRP Conjugate, #1721011, Bio-rad) diluted 1:4,000. The immunoreaction was developed with a chemiluminescent method based on luminol. Quantification of band intensity was carried out with Quantity One 1-D analysis software (Bio-Rad). The ratio of the signal intensity of VEGF-A versus the loading control was calculated. Within each blot, values are expressed as fold vs. mean non-ischemic control tissue.

***Immunohistochemistry of paraffin embedded sections***

Rats were anesthetized with isoflurane and perfused through the heart with saline containing 0.1% heparin, followed by 4% paraformaldehyde. The brain was fixed overnight with the same fixative, washed with phosphate buffer pH7.4, embedded in paraffin and cut into 5 μm-thick sections with a microtome. Immunohistochemistry was performed with the following primary antibodies: a mouse monoclonal antibody against CD163 (clone ED2) (#MCA342GA, AbD Serotec, Bio-Rad) diluted 1:50, and rabbit polyclonal antibodies against Iba-1 (#019-19741, Wako Chemicals USA, Inc.) diluted 1:500, and against myeloperoxidase (MPO) (#A0398, Dako), diluted 1:100, using the EnVisionTM Detection System; (#K5007, Dako). The sections were deparaffinized, and if necessary (for CD163 and Iba-1) they were subjected to antigen retrieval using the EnVision buffers in a microwave for 10 min. For endogenous peroxidase activity blocking, the sections were immersed in PBS containing 1% hydrogen peroxide and 30% methanol for 15 min. The sections were pre-incubated with corresponding normal serum (3%) for 1 h and then incubated overnight at 4ºC with the primary antibody. Sections were then incubated with secondary biotinylated antibodies diluted 1:200 for 2 hours at RT. Sections were then processed with the avidin-biotin peroxidase method (ABC, Vector, Palex Medical S.A., Sant Cugat del Vallès, Spain). The immunoreaction was visualized with the diamenobenzidine technique and the sections were lightly counterstained with hematoxylin and examined under the optical microscope. The number of cells per area was counted in three large regions of interest (ROI areas measured between 1 and 2 mm^2^) of the cortex and the striatum in two paraffin sections per rat. A researcher blind to the treatments obtained brain images using the 40x objective of a microscope (Olympus BX51) with motorized stage (Prior Pro Scan II) and equipped with a digital camera (Olympus DP71). Cell counting was performed using stereology software (Visiopharm Integrator System, newCAST™ version 3.2.4.0, Visiopharm, Hoersholm, Denmark). The average of the cell counts measured in the different ROIs was calculated per each region. The process was independently repeated twice and the average of the two measures was taken. Values are expressed as mean number of cells per mm^2^.

***RNA microarray and bioinformatics***

Six RNA samples obtained from the sorted CD11b^+^CD163^+^ cells (from three control rats and three ischemic rats at 16h post-ischemia) with RIN values>9.2 were selected for RNA microarray study with GeneChip Rat Genome 230 2.0 Array, 3’IVT Pico reagent kit (Affymetrix). The images were processed with the Expression Console software (Affymetrix) to check the array quality. All samples demonstrated good quality results (pm mean, bgrd mean, pos vs neg auc) and were subjected to subsequent analysis. Normalization was applied to array data to correct for systematic bias not related to biological variation. In order to make the data comparable and to remove technical biases these arrays were pre-processed using the RMA method [4]. These normalized values were the basis for all the analysis. Prior to any analysis, data were subjected to non-specific filtering to remove low signal genes and low variability genes. The selection of differentially expressed genes between conditions was based on a linear model analysis with empirical Bayes moderation of the variance estimates as described [8]. *P*-values were adjusted to obtain strong control over the false discovery rate using the Benjamini and Hochberg method [1]. Genes significantly different between groups were graphically highlighted using ‘volcano plots’. Genes selected as being differentially expressed were clustered to look for common patterns of expression. Hierarchical clustering with euclidean distance was used to form the groups and heatmaps were used for visualization. The analysis of Biological Significance was based on an overrepresentation analysis aiming at establishing if the genes that were found to be differentially expressed appeared to be concentrated or particularly absent of some Gene Ontology (GO) categories. All the statistical analyses were carried out using the free statistical language R and the libraries developed for microarray data analysis by the Bioconductor Project ([www.bioconductor.org](http://www.bioconductor.org)). The functional analyses were generated through the use of DAVID Bioinformatics Resources 6.7 [2,3], and Ingenuity Pathway Analysis (IPA, QIAGEN Inc., <https://www.qiagenbioinformatics.com/products/ingenuity-variant-analysis/>) [5].

**REFERENCES**

1. Benjamini Y, Hochberg Y (1995) Controlling the false discovery rate: A practical and powerful approach to multiple testing. J Royal Stat Soc (Series B, Methodological) 57:289-300

2. Huang DW, Sherman BT, Lempicki RA (2009) Systematic and integrative analysis of large gene lists using DAVID Bioinformatics Resources. Nature Protoc 4:44-57

3. Huang DW, Sherman BT, Lempicki RA (2009) Bioinformatics enrichment tools: paths toward the comprehensive functional analysis of large gene lists. Nucleic Acids Res 37:1-13

4. Irizarry RA, Hobbs B, Collin F, Beazer-Barclay YD, Antonellis KJ, Scherf U, et al. (2003) Exploration, normalization, and summaries of high density oligonucleotide array probe level data. Biostatistics 4:249-64

5. Krämer A, Green J, Pollard J Jr, Tugendreich S (2014) Causal analysis approaches in Ingenuity Pathway Analysis. Bioinformatics 30:523-30

6. Pérez-Asensio FJ, de la Rosa X, Jiménez-Altayó F, Gorina R, Martínez E, Messeguer A, et al. (2010) Antioxidant CR-6 protects against reperfusion injury after a transient episode of focal brain ischemia in rats. J Cereb Blood Flow Metab 30:638-52

7. Polfliet MM, Goede PH, van Kesteren-Hendrikx EM, van Rooijen N, Dijkstra CD, van den Berg TK (2001) A method for the selective depletion of perivascular and meningeal macrophages in the central nervous system. J Neuroimmunol 116:188-95

8. Smyth GK (2004) Linear models and empirical bayes methods for assessing differential expression in microarray experiments. Stat Appl Genet Mol Biol 3:Article3

9. Soria G, Tudela R, Márquez-Martín A, Camón L, Batalle D, Muñoz-Moreno E, et al. (2013) The ins and outs of the BCCAo model for chronic hypoperfusion: a multimodal and longitudinal MRI approach. PLoS One 8:e74631

10. Soriano MA, Sanz O, Ferrer I, Planas AM (1997) Cortical infarct volume is dependent on the ischemic reduction of perifocal cerebral blood flow in a three-vessel intraluminal MCA occlusion/reperfusion model in the rat. Brain Res 747:273-8

**SUPPLEMENTARY TABLES**

**Table S1.**

**Top 25 genes upregulated in CD163^+^ BAMs 16h post-ischemia vs. control**

|  |  |  |  |  | **Control** | |  |  | **Ischemia** | |  |
| --- | --- | --- | --- | --- | --- | --- | --- | --- | --- | --- | --- |
| **Gene Symbols** | **logFC** | **P.Value** | **adj.P.Val** | **1** | **2** | **3** | | **4** | | **5** | **6** |
| **Vcan** | 8.134 | 3.48E-10 | 1.25E-06 | 4.110 | 4.480 | 4.050 | | 11.836 | | 12.390 | 12.816 |
| **Sdc1** | 7.212 | 1.01E-10 | 7.31E-07 | 5.191 | 5.599 | 5.261 | | 12.333 | | 12.532 | 12.823 |
| **Arg1** | 6.523 | 1.06E-07 | 6.98E-05 | 5.588 | 7.072 | 7.158 | | 12.752 | | 13.046 | 13.589 |
| **Tgm2** | 5.990 | 1.74E-06 | 2.17E-04 | 7.263 | 5.876 | 6.234 | | 11.198 | | 13.084 | 13.061 |
| **Fn1** | 5.963 | 1.37E-06 | 1.93E-04 | 8.571 | 7.586 | 6.386 | | 12.785 | | 13.927 | 13.721 |
| **Enpp3** | 5.613 | 3.71E-08 | 4.45E-05 | 7.419 | 7.449 | 7.243 | | 12.273 | | 13.032 | 13.646 |
| **Slpi** | 5.359 | 5.93E-05 | 1.19E-03 | 10.111 | 6.591 | 7.434 | | 13.110 | | 13.492 | 13.611 |
| **Sema3c** | 5.253 | 1.23E-06 | 1.85E-04 | 6.891 | 5.554 | 5.449 | | 10.451 | | 11.356 | 11.845 |
| **Upp1** | 5.119 | 9.61E-07 | 1.74E-04 | 7.255 | 6.543 | 6.226 | | 10.809 | | 12.330 | 12.243 |
| **Spp1** | 5.093 | 2.46E-08 | 3.55E-05 | 9.278 | 9.358 | 10.289 | | 14.676 | | 14.702 | 14.826 |
| **Il6** | 4.832 | 1.93E-06 | 2.31E-04 | 5.168 | 4.775 | 6.579 | | 10.760 | | 10.327 | 9.930 |
| **Mmp14** | 4.789 | 7.40E-08 | 6.21E-05 | 7.658 | 7.618 | 7.554 | | 11.734 | | 12.489 | 12.972 |
| **Mmp7** | 4.681 | 5.98E-06 | 4.26E-04 | 5.293 | 5.252 | 5.275 | | 8.610 | | 10.743 | 10.510 |
| **Chi3l1** | 4.635 | 1.63E-05 | 6.62E-04 | 7.120 | 7.113 | 6.417 | | 10.086 | | 12.498 | 11.970 |
| **LOC100910286** | 4.500 | 2.75E-07 | 1.06E-04 | 7.534 | 6.741 | 7.727 | | 11.308 | | 12.259 | 11.933 |
| **Procr** | 4.239 | 3.84E-07 | 1.17E-04 | 6.372 | 5.782 | 6.534 | | 9.833 | | 10.933 | 10.637 |
| **Anpep** | 4.234 | 5.62E-06 | 4.05E-04 | 7.902 | 7.731 | 7.758 | | 10.902 | | 12.269 | 12.923 |
| **Slc47a1** | 4.160 | 2.98E-06 | 3.00E-04 | 5.568 | 5.873 | 6.542 | | 9.439 | | 10.038 | 10.986 |
| **Bcat1** | 4.119 | 2.71E-07 | 1.06E-04 | 8.169 | 8.418 | 7.786 | | 11.617 | | 12.525 | 12.587 |
| **Dusp2** | 3.999 | 9.95E-07 | 1.75E-04 | 9.049 | 8.858 | 8.205 | | 12.009 | | 13.029 | 13.071 |
| **Ccl17** | 3.984 | 1.67E-06 | 2.17E-04 | 8.547 | 8.185 | 8.016 | | 11.427 | | 12.931 | 12.342 |
| **G0s2** | 3.931 | 1.77E-07 | 1.06E-04 | 8.159 | 8.378 | 8.126 | | 11.543 | | 12.444 | 12.469 |
| **Ass1** | 3.900 | 3.76E-04 | 2.97E-03 | 5.058 | 5.621 | 5.655 | | 7.438 | | 10.707 | 9.890 |
| **Bst1** | 3.883 | 9.23E-07 | 1.74E-04 | 6.684 | 6.293 | 6.533 | | 9.661 | | 10.485 | 11.014 |
| **Gjb2** | 3.772 | 2.10E-05 | 7.53E-04 | 6.822 | 6.191 | 5.908 | | 8.944 | | 10.778 | 10.515 |

**Table S2.**

**Top 25 genes downregulated in CD163^+^ BAMs 16h post-ischemia vs. control**

|  |  |  |  |  | **Control** | |  |  | **Ischemia** | | |  |
| --- | --- | --- | --- | --- | --- | --- | --- | --- | --- | --- | --- | --- |
| **Gene Symbols** | **logFC** | **P.Value** | **adj.P.Val** | **1** | | **2** | **3** | **4** | | **5** | **6** | |
| **Gria2** | -3.75 | 8.09E-05 | 1.39E-03 | 7.502 | | 5.560 | 6.207 | 10.198 | | 9.281 | 12.816 | |
| **Fam3b** | -3.64 | 1.03E-05 | 5.76E-04 | 7.174 | | 5.933 | 5.996 | 9.291 | | 10.217 | 12.823 | |
| **Wdr95** | -3.42 | 1.18E-05 | 5.99E-04 | 7.229 | | 6.653 | 7.882 | 10.225 | | 10.380 | 13.589 | |
| **Fam65b** | -3.41 | 4.60E-05 | 1.07E-03 | 8.843 | | 7.458 | 6.725 | 10.883 | | 11.061 | 13.061 | |
| **Itgb5** | -3,16 | 9.23E-06 | 5.59E-04 | 8.356 | | 7.409 | 6.917 | 10.439 | | 11.000 | 13.721 | |
| **Cd7** | -3.12 | 8.29E-06 | 5.24E-04 | 8.575 | | 7.777 | 7.120 | 11.176 | | 10.763 | 13.646 | |
| **Slc18b1** | -3.04 | 8.65E-05 | 1.42E-03 | 9.304 | | 7.203 | 8.468 | 11.452 | | 11.315 | 13.611 | |
| **Slc16a12** | -2.97 | 3.85E-06 | 3.47E-04 | 7.751 | | 7.129 | 6.792 | 9.820 | | 10.164 | 11.845 | |
| **Igh-6** | -2.94 | 1.18E-06 | 1.85E-04 | 8.169 | | 7.591 | 7,209 | 10.594 | | 10.690 | 12.243 | |
| **Egfl7** | -2.87 | 8.52E-07 | 1.70E-04 | 9.317 | | 8.738 | 8.842 | 11.474 | | 12.001 | 14.826 | |
| **Tnfrsf25** | -2.82 | 6.41E-05 | 1.22E-03 | 8.007 | | 6.563 | 6.678 | 9.437 | | 10.354 | 9.930 | |
| **Tspan8** | -2.79 | 3.90E-04 | 3.03E-03 | 9.712 | | 7.731 | 7.608 | 10.823 | | 11.344 | 12.972 | |
| **Negr1** | -2.77 | 1.07E-04 | 1.57E-03 | 7.370 | | 6.290 | 5.895 | 8.576 | | 9.592 | 10.510 | |
| **Syne2** | -2.73 | 4.06E-05 | 1.01E-03 | 8.555 | | 7.570 | 6.990 | 10.285 | | 10.717 | 11.970 | |
| **Dnase1l3** | -2.73 | 4.40E-05 | 1.06E-03 | 6.375 | | 5.624 | 5.772 | 7.829 | | 9.252 | 11.933 | |
| **Hacd4** | -2.71 | 2.91E-04 | 2.54E-03 | 10.139 | | 8.270 | 8.390 | 11.180 | | 11.922 | 10.637 | |
| **Siglec5** | -2.69 | 7.21E-04 | 4.40E-03 | 11.354 | | 9.892 | 8.902 | 12.328 | | 13.082 | 12.923 | |
| **Igfbp4** | -2.68 | 1.13E-05 | 5.98E-04 | 7.985 | | 7.705 | 8.340 | 10.036 | | 10.984 | 10.986 | |
| **Gpr155** | -2.68 | 1.12E-05 | 5.98E-04 | 7.483 | | 6.460 | 6.932 | 9.173 | | 9.906 | 12.587 | |
| **Ermn** | -2.68 | 1.14E-03 | 5.72E-03 | 4.799 | | 4.864 | 6.732 | 9.080 | | 7.881 | 13.071 | |
| **LOC102549097** | -2.65 | 1.17E-05 | 5.99E-04 | 8.419 | | 7.835 | 7.465 | 10.113 | | 10.976 | 12.342 | |
| **Egfr** | -2.62 | 2.50E-05 | 8.22E-04 | 8.309 | | 7.316 | 7.292 | 9.758 | | 10.530 | 12.469 | |
| **Adam23** | -2.59 | 7.88E-05 | 1.38E-03 | 9.103 | | 7.931 | 8.230 | 11.562 | | 11.107 | 9.890 | |
| **Deptor** | -2.57 | 7.58E-05 | 1.35E-03 | 9.274 | | 7.783 | 8.168 | 10.576 | | 11.128 | 11.014 | |
| **LOC102547294** | -2.54 | 5.43E-04 | 3.68E-03 | 6.180 | | 5.675 | 6.354 | 7.410 | | 8.844 | 10.515 | |

**

**

**Fig. S1 Flow cytometry characterization of sorted cells**

Gating strategy for cell sorting and subsequent purity check of the sorted cells by flow cytometry. Cells obtained from the control brain (a) or the ischemic brain tissue 16h after induction of MCAo (b) were labeled with CD11b and CD163 antibodies. Cells were sorted separately in CD11b^+^CD163^+^ cells (R1) and CD11b^+^CD163^-^ cells (R2) according to the gates shown in the plots (third plot in the top row of panels A and B). The purity of the sorted cells was checked by flow cytometry and it is illustrated in the plots. The CD11b^+^CD163^+^ cells correspond to BAMs (plots shown in the right hand side of the first raw in A and B) and they are mostly CD45^hi^CD11b^+^ cells, both in the control brain and the ischemic brain. The sorted CD163^+^ cells contained 5% of REA^+^ granulocytes in the ischemic brain tissue. For the microglia gate (R2) we took CD163^-^ CD11b^+^ cells expressing low levels of CD11b to ensure that we did not include CD11b^hi^ cells that might correspond to infiltrating myeloid cells. Accordingly, flow cytometry after cell sorting showed that the CD163^-^ sorted cells were mostly CD45^dim^ microglia and contained negligible amounts of CD11b^+^ CD45^hi^ infiltrating myeloid cells or REA535^+^ granulocytes (bottom raw in panels a and b).

**
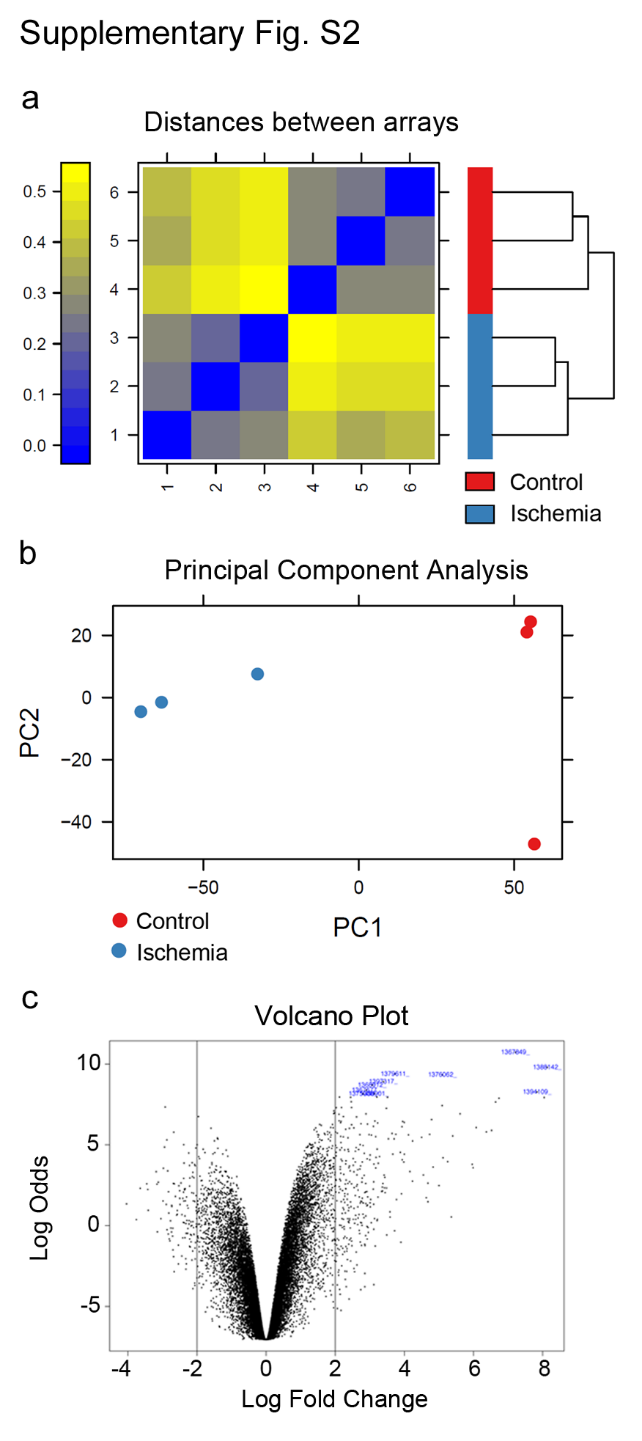
**

**Fig. S2 Descriptive data of the Affymetrix microarray analysis of RNA from sorted CD163^+^ macrophages**

a) The heatmap of the distances between arrays show clustering according to the condition (ischemia vs. control, n=3 per group). The distance dab between two arrays a and b is computed as the mean absolute difference (L1-distance) between the data of the arrays (using the data from all probes without filtering). In formula, dab = mean | Mai - Mbi |, where Mai is the value of the i-th probe on the a-th array. Outlier detection was performed by looking for arrays for which the sum of the distances to all other arrays, Sa = Σb dab was exceptionally large. No outlier arrays were detected in the sample. b) The image shows the Principal Components (PC) where arrays cluster according to groups (control vs. ischemia). PC1 explains most of the variability of the sample, 76%, and perfectly separates ischemic samples from control samples. PC2 explains 11% of the variability and illustrates differences between animals. c) Microarray analysis with LogFC>2 and FDR<0.01 showed that the expression of 1493 genes was up-regulated whereas the expression of 594 genes was down-regulated in CD163^+^ macrophages 16h after ischemia, as graphically illustrated in the Volcano plot.

**
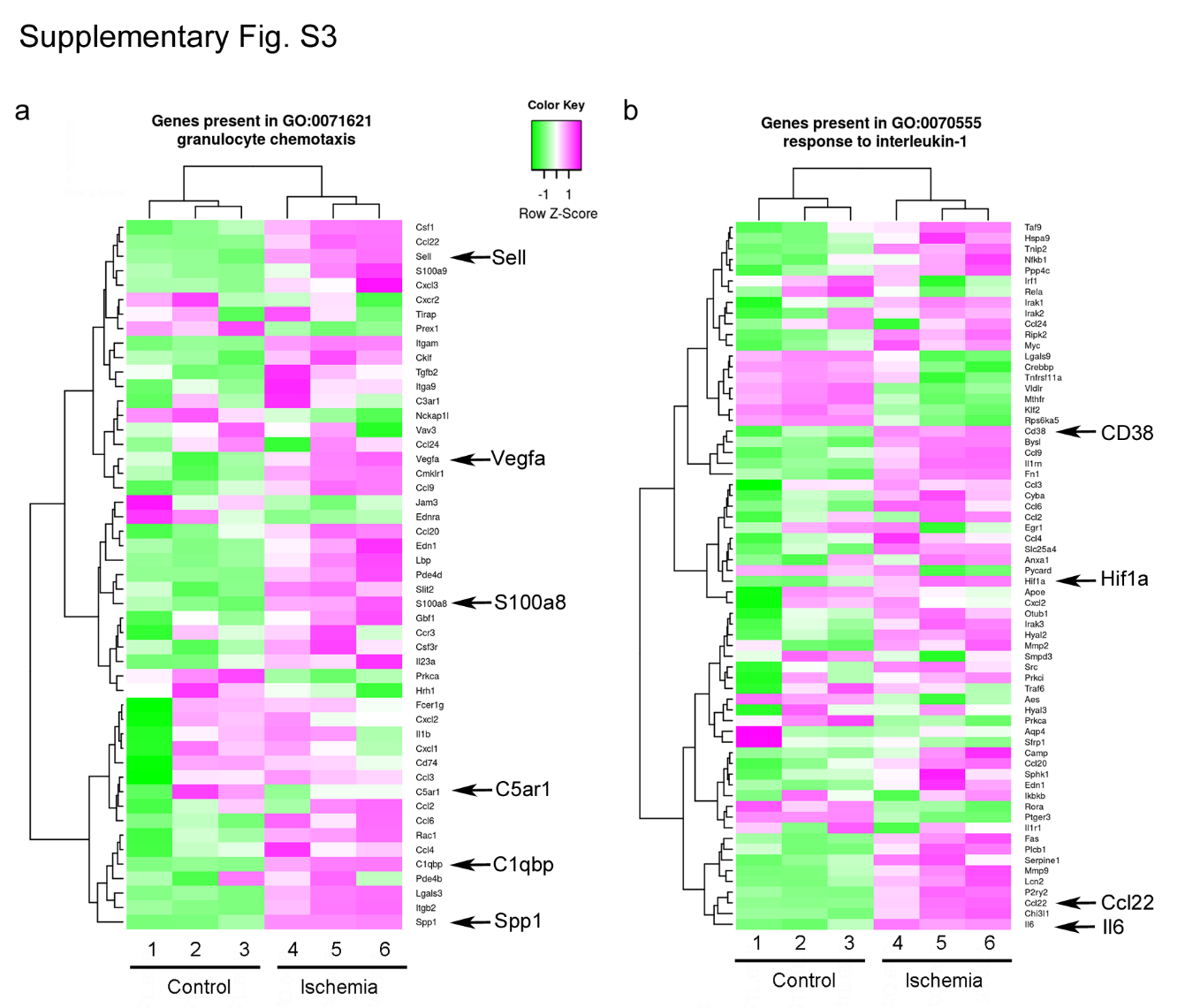
**

**Fig. S3 Heatmaps for genes present in significant GO terms**

RNA of CD163^+^ macrophages from ischemic rats (16h post-ischemia) versus controls (n=3 per group). a) Granulocyte chemotaxis (GO:0071621). b) Response to Interleukin-1 (GO:0070555). Upregulated genes are shown in pink and down-regulated genes appear in green (see color-code scale in the top center of the image). Arrows indicate some of the genes mentioned in the main text.

**
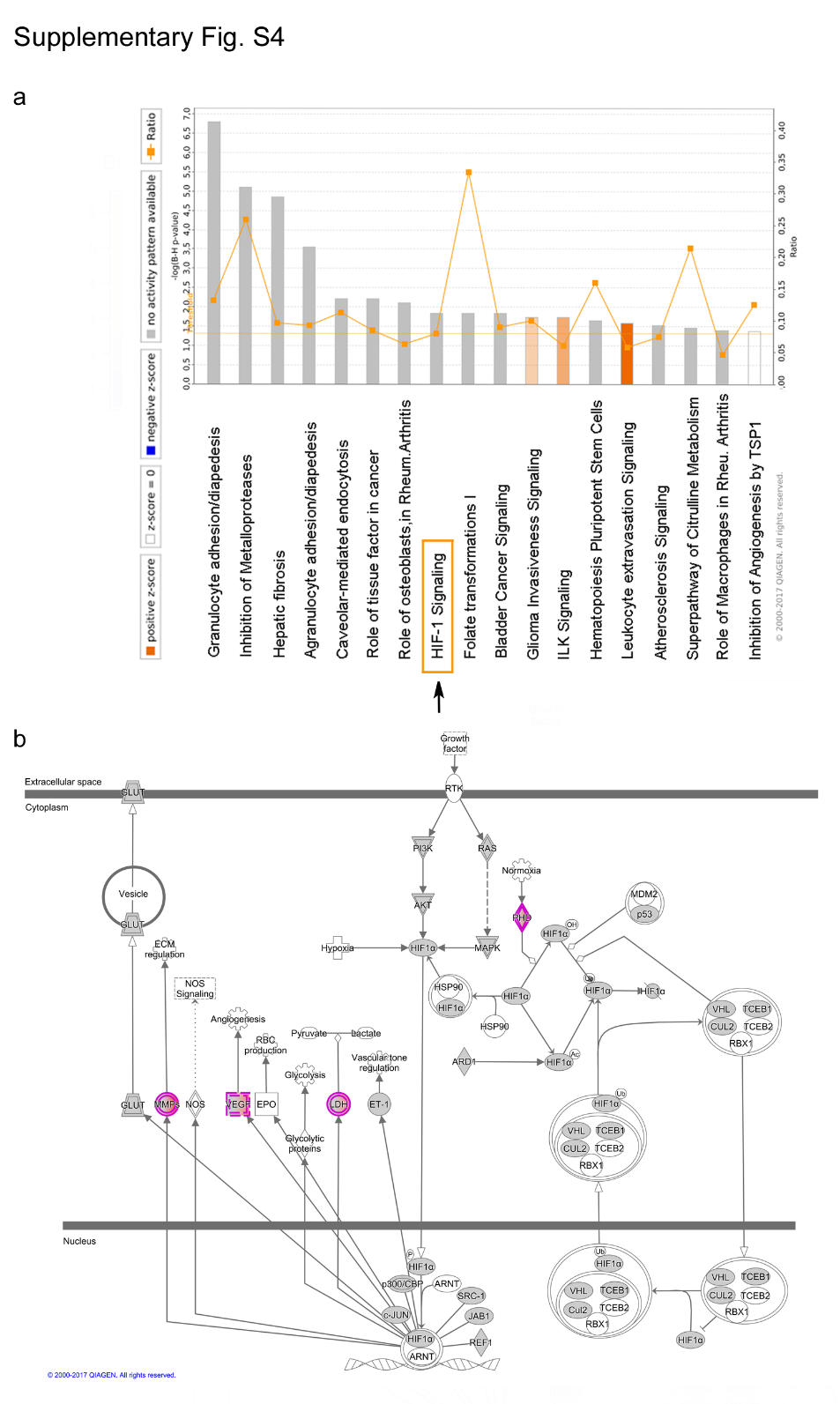
**

**Fig. S4 Biological significance of gene expression in CD163^+^ macrophages based on enrichment analysis 16h after ischemia versus controls**

a) Top canonical pathways derived from Ingenuity pathway analysis (IPA). The Ratio is calculated by dividing the number of molecules from our dataset that map to each pathway by the total number of molecules in the canonical pathway of the IPA knowledgebase (yellow line). Hypoxia-inducible factor (HIF-1) signalling is indicated with an arrow. b) IPA Network for HIF-1 shows in pink highly upregulated nodes in CD163^+^ macrophages sorted after ischemia versus control brain.


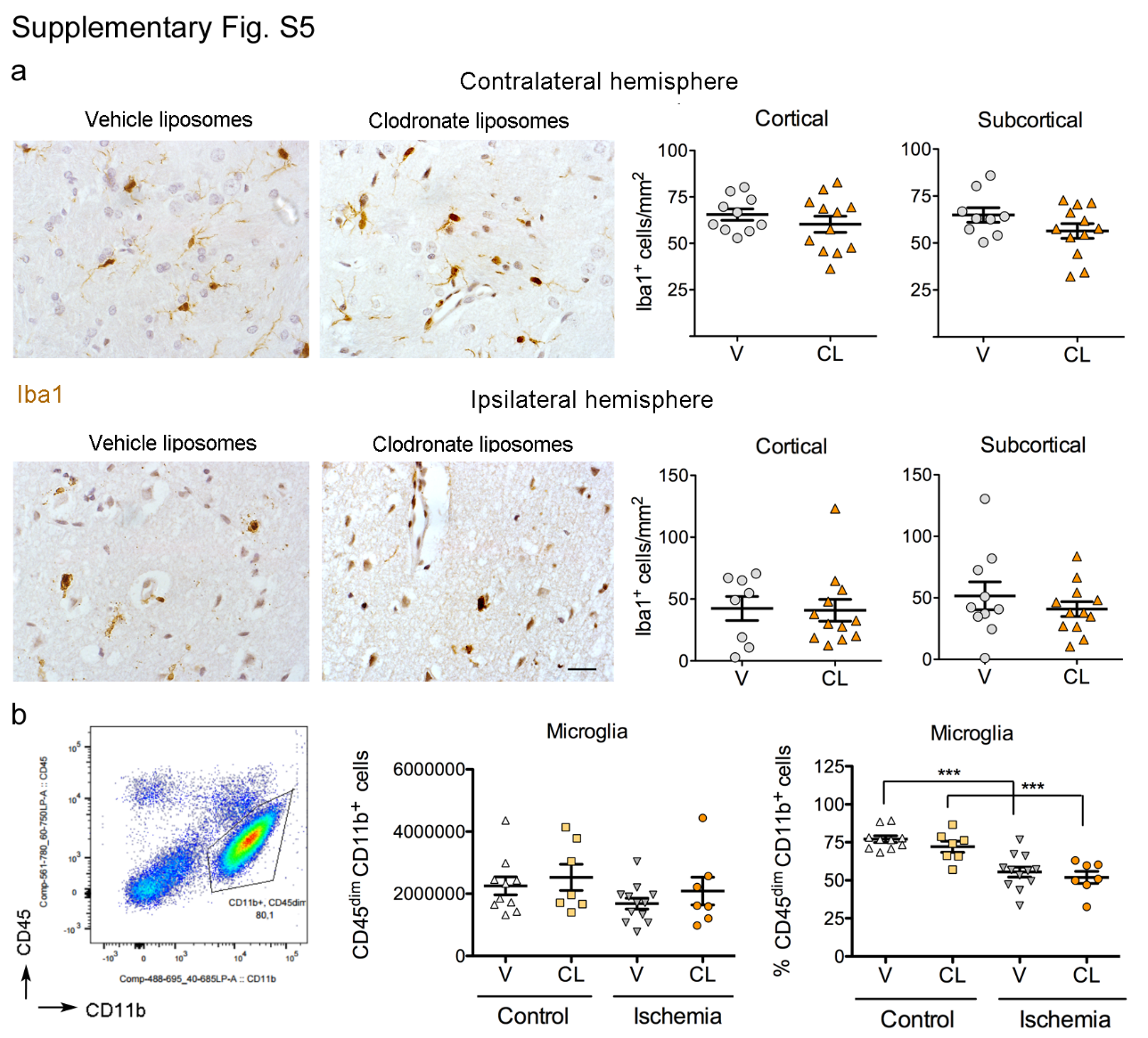


**Fig. S5 Clodronate administration does not alter microglial cell number**

Rats received clodronate (CL) or vehicle (V) liposomes in the left ventricle 4 days before occlusion of the right MCA and the brain was analyzed 1 day after ischemia. a) CL (n=12) does not alter the numbers of microglial cells in the contralateral and ipsilateral (ischemic) hemispheres versus the V group (n=10), as assessed in cortical and subcortical regions by Iba-1 immunohistochemistry and cell counting. Cell counting was performed within the core of infarction and corresponding zones of the contralateral hemisphere. Some Iba1^+^ cells are seen in perivascular spaces after clodronate in both hemispheres. b) Flow cytometry analysis of CD45^dim^CD11b^+^ cells (microglia) in controls (n=11 vehicle, V; n=9 CL) and 16h post-ischemia (n=12 V; n=11 CL) using the whole brain hemisphere showed no significant diferences in microglia cell numbers after treatment. Ischemia reduced the percentage of microglial cells in both treatment groups. Two-way ANOVA by condition (ischemia vs. control) and treatment, *** p<0.001. Scale bar: 20 μm.


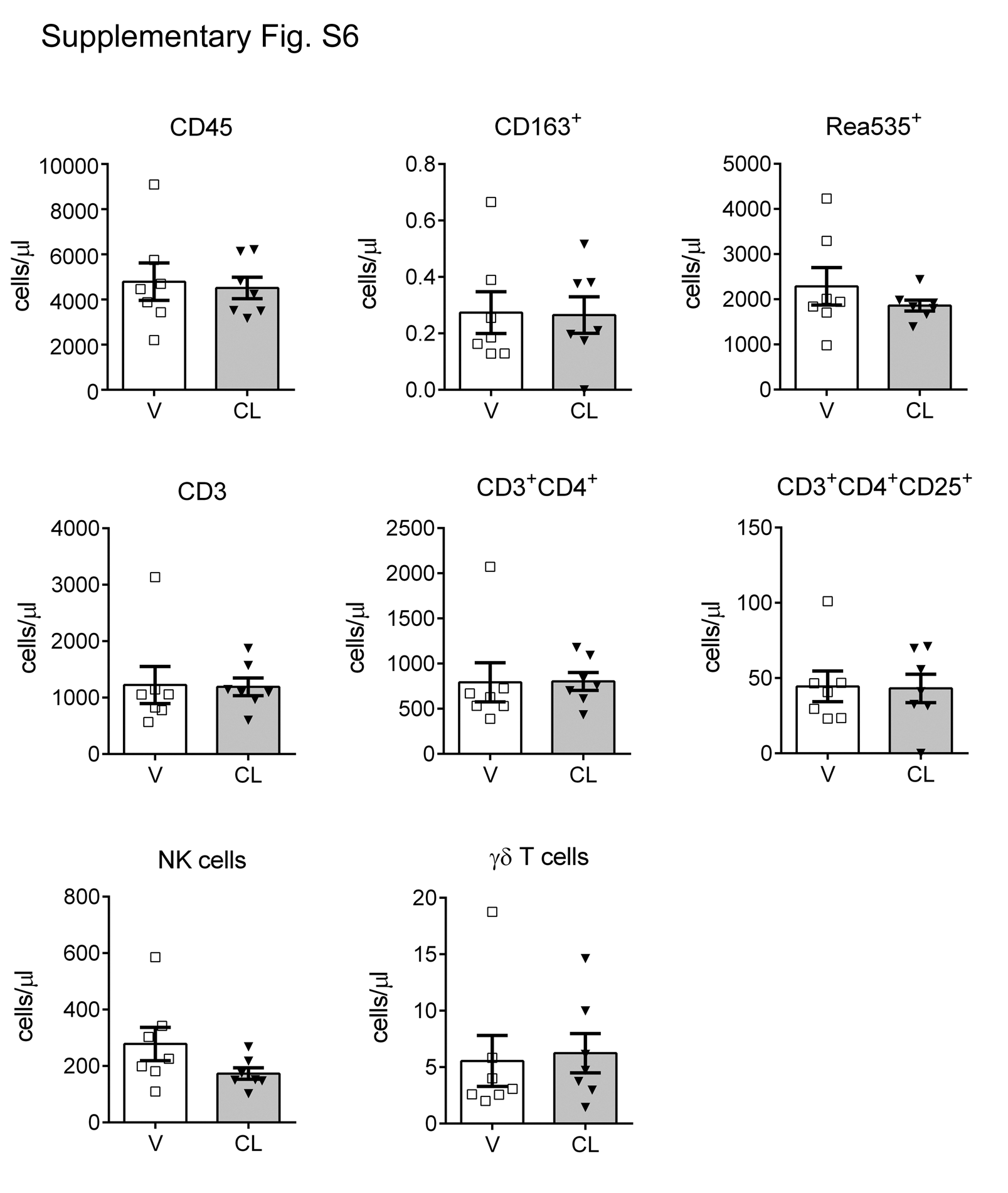


**Fig. S6 Intracerebroventricular administration of clodronate does not alter blood cell number**

Rats received adminsitration of liposomes containing either clodronate (CL) or the vehicle (V) in the left ventricle (n=7 per group). Four days later, ischemia was induced by 1-hour occlusion of the right MCA. The blood was studied one day later by flow cytometry to quantify the different cell populations. Local brain treatment did not modify the numbers of circulating cells.


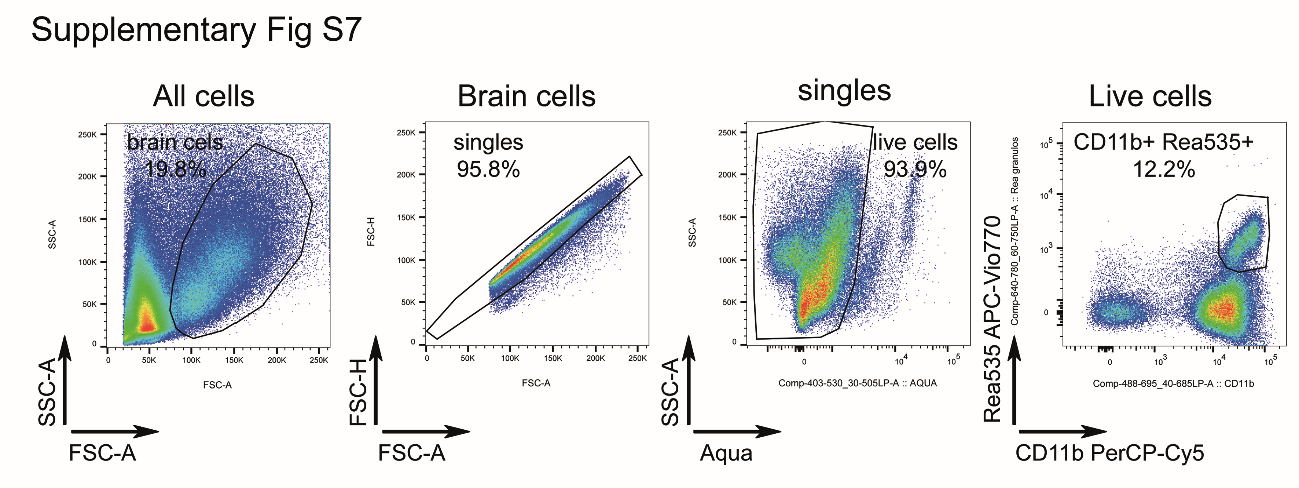


**Fig. S7 Gating strategy for flow cytometry of granulocytes in the ischemic brain tissue**

Single-cell suspensions of brain tissue were obtained with a Neural dissociation kit followed by percoll gradient. Cells were stained with Aqua life/death staining, followed by immunostaining with antibodies against CD11b and REA535.


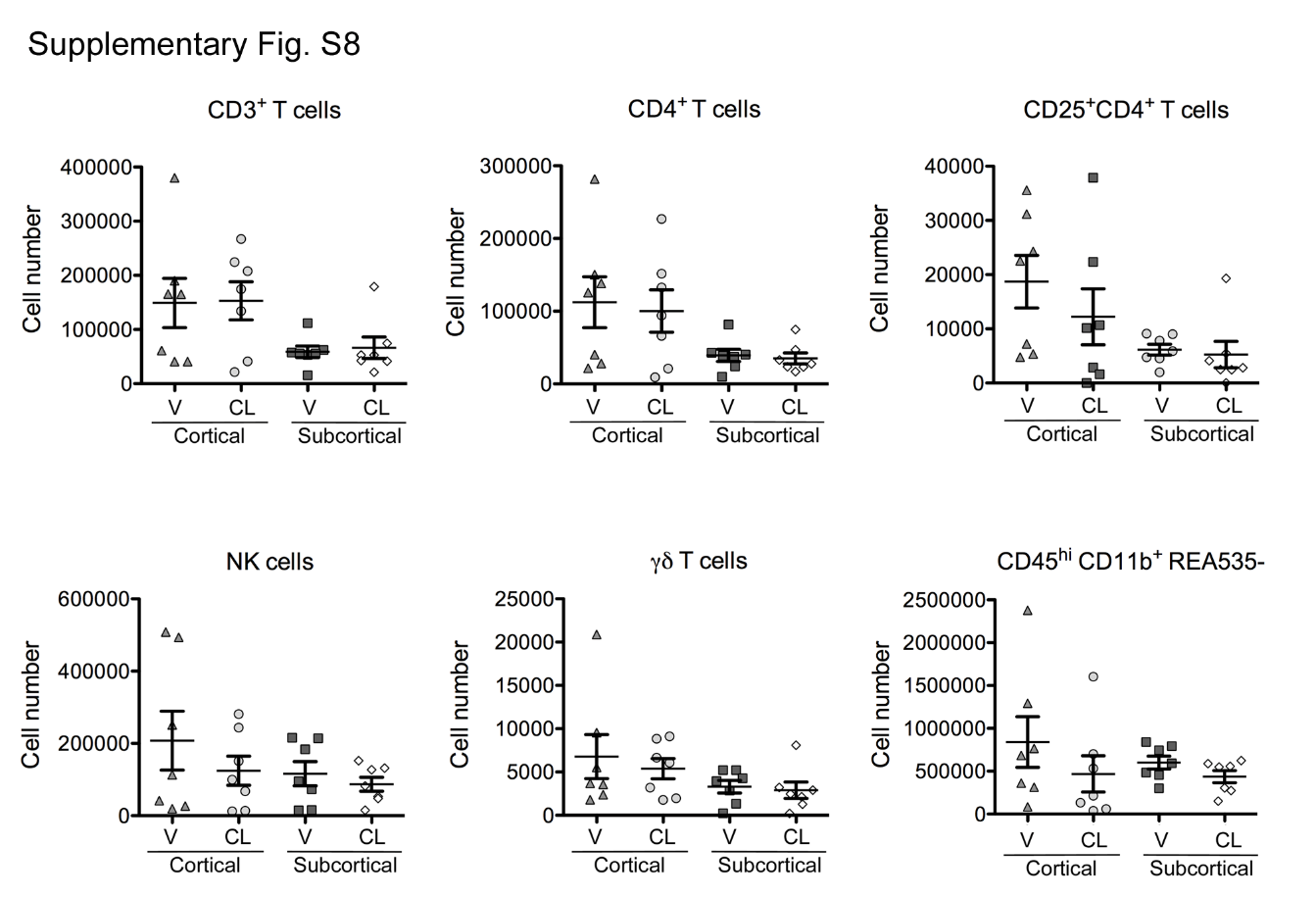


**Fig. S8 BAM depletion did not modify lymphocyte or myeloid mononuclear cell infiltration in the ischemic brain tissue**

Rats received clodronate (CL) or vehicle (V) liposomes in the left ventricle 4 days before occlusion of the right MCA and the brain was analyzed 1 day after ischemia (n=7 per group). Clodronate treatment did not significantly (Mann-Whitney test) modify the numbers of CD3^+^ T cells, CD4^+^ T cells, CD25^+^CD4^+^ T cells, NK cells, γδ T cells, or myeloid mononuclear cells (CD45^hi^ CD11b^+^ REA535^-^) infiltrated in cortical or subcortical ischemic brain regions. Values are expressed as number of cells/g of tissue.
